# Recursive integration of synergised graph representations of multi-omics data for cancer subtypes identification

**DOI:** 10.1038/s41598-022-17585-2

**Published:** 2022-09-17

**Authors:** Madhumita, Archit Dwivedi, Sushmita Paul

**Affiliations:** 1 Department of Bioscience and Bioengineering, Indian Institute of Technology, Jodhpur, Rajasthan 342037 India; 2 School of Artificial Intelligence and Data Science, Indian Institute of Technology, Jodhpur, Rajasthan 342037 India

**Keywords:** Cancer, Computational biology and bioinformatics

## Abstract

Cancer subtypes identification is one of the critical steps toward advancing personalized anti-cancerous therapies. Accumulation of a massive amount of multi-platform omics data measured across the same set of samples provides an opportunity to look into this deadly disease from several views simultaneously. Few integrative clustering approaches are developed to capture shared information from all the views to identify cancer subtypes. However, they have certain limitations. The challenge here is identifying the most relevant feature space from each omic view and systematically integrating them. Both the steps should lead toward a global clustering solution with biological significance. In this respect, a novel multi-omics clustering algorithm named RISynG (Recursive Integration of Synergised Graph-representations) is presented in this study. RISynG represents each omic view as two representation matrices that are Gramian and Laplacian. A parameterised combination function is defined to obtain a synergy matrix from these representation matrices. Then a recursive multi-kernel approach is applied to integrate the most relevant, shared, and complementary information captured via the respective synergy matrices. At last, clustering is applied to the integrated subspace. RISynG is benchmarked on five multi-omics cancer datasets taken from The Cancer Genome Atlas. The experimental results demonstrate RISynG’s efficiency over the other approaches in this domain.

## Introduction

Cancer is a heterogeneous disease with diverse pathogeneses, and clinical features that can develop in different tissues and cell types^1^. A cancer subtype can be defined as a subcategory of specific cancer; for example, Cervical cancer can be further grouped into Adenocarcinomas and Squamous cell carcinomas. Multiple subtypes are distinguishable based on molecular profiles, histology, or sometimes specific mutation. In personalized medicine practices, patient-specific medicines are provided rather than generic medicine. Therefore, for effective treatment of any cancer, it is crucial to identify the appropriate cancer subtype in order to provide an effective prognosis^2^.

Nowadays, with the advancement of technologies, it has become very easy to generate high-dimensional multi-omics data for an individual. Multi-omics data include miRNA and mRNA expressions, DNA methylation, reverse protein phase assays, and others. These datasets are publicly available in various databases like The Cancer Genome Atlas (TCGA)^3^. Accumulation of various omics data opens up the opportunity to develop novel computational methods to integrate the tremendous amount of multi-view information available for cancer subtype identification. The usual practice of identifying cancer subtypes is by clustering cancer patient data. By grouping the cancer patients based on their genetic profiles, one can better understand the pathogenic mechanisms behind the disease. This will later help in the development of subtype-specific anticancer treatments. However, several challenges exist in grouping the cancer patients and integrating multi-omics data.

The multi-view omics data integration and clustering of cancer patients are considerably new research areas. Few algorithms are developed to address the challenges associated with it. A decade ago, researchers used single omics data to cluster cancer subtypes. Several studies are performed using only gene expression data^4–6^ or DNA methylation data^7^ or copy number data^8^ to identify cancer subtypes. These algorithms perform clustering across the samples to capture the homogeneity present within the patients based on expression levels of a specific biomarker. Since acquiring cancer hallmarks requires multiple molecular alterations at multiple levels, these algorithms fail to establish the causal relationship between molecular signatures. This biological phenomenon indicates the need for algorithms that integrates multi-omics data to identify cancer subtype. In this regard, integrative clustering-based approaches are found helpful for capturing underlying molecular mechanisms working behind deadly cancer. Further, these algorithms can be categorized into two groups. The first group of algorithms identifies clusters from each omic data separately. Later, it combines these clustering results to obtain a global cluster that represents cancer subtypes^9–12^. These forms of algorithms are known as Consensus Clustering (CC). Mostly, the CC algorithms perform final clustering on individual clusters obtained from different omic datasets using a voting mechanism. Different voting mechanisms generate different clustering solutions. The second group of integrative clustering-based approaches first integrates the multi-view omics data and then applies clustering to obtain cancer subtypes^13–16^. Sometimes the multi-view data are concatenated or stacked together, and clustering identifies cancer subtypes. Data concatenation may lead to information loss and amplifies the curse of dimensionality^16^. On the other hand, to overcome the above mentioned limitations, a set of algorithms are developed to extract informative subspace from each of the omics datasets and then performs clustering on the integrated dataset^14–19^.

Clustering multi-view genomics data is a challenging task. One of the critical steps is selecting relevant information from all the available information sources and judiciously integrating them to obtain better clustering solutions. The multi-view data from multi-omics studies vary in terms of variance, scale, and unit. If the integration step is not performed correctly, the fused information may be biased towards the most variant omic view. Therefore, it becomes essential to first capture the variations present in each view and then integrate them. There are some methods available that model the variation of each view first with the help of similarity graphs and integrate them to identify clusters^13,19–21^. The challenge here is finding the best possible way of integration to capture the essence of all the views from different types of genomic information available for the same set of samples. The research area devoted to this type of problem is multi-view learning^22–27^.

In this study, a novel algorithm named RISynG (Recursive Integration of Synergised Graph-representations) is presented. The proposed approach treats multi-omics data clustering as multi-view clustering, where information from multiple omics platforms is integrated to identify clinically important sub-groups within cancer. In order to judiciously capture the variation present across the multi-omics dataset, the proposed approach works in three steps. At first, for each view, two sample similarity matrices are computed using graph representation matrices, namely, the Gramian matrix and the Laplacian matrix. This step acknowledges the statistical diversity in the multi-view omics data, which directly influences the quantification of similarity between the samples. Later, it involves the integration of representation matrices for the respective omic-view using a parameterized combination function to generate synergy matrices. In the second step, the variation captured through synergy matrices for each omic-view is fused. The proposed approach first arranges all the synergy matrices based on their relevance. Then, a recursive function is designed to merge each synergy matrix so that the less relevant matrix has only a slight influence on the final cluster structures. At the end of this process, the final accretive basis of the accretive subspace is obtained, whose first *k* eigenvectors hold the cluster structure. At last, *k*-means clustering is applied on the rows of the accretive basis matrix to generate cluster labels. The efficacy of the proposed algorithm is extensively studied on five multi-omics cancer datasets and compared with existing multi-view clustering approaches used for cancer subtypes identification.

## Proposed approach for cancer-subtypes identification

**Figure 1:**
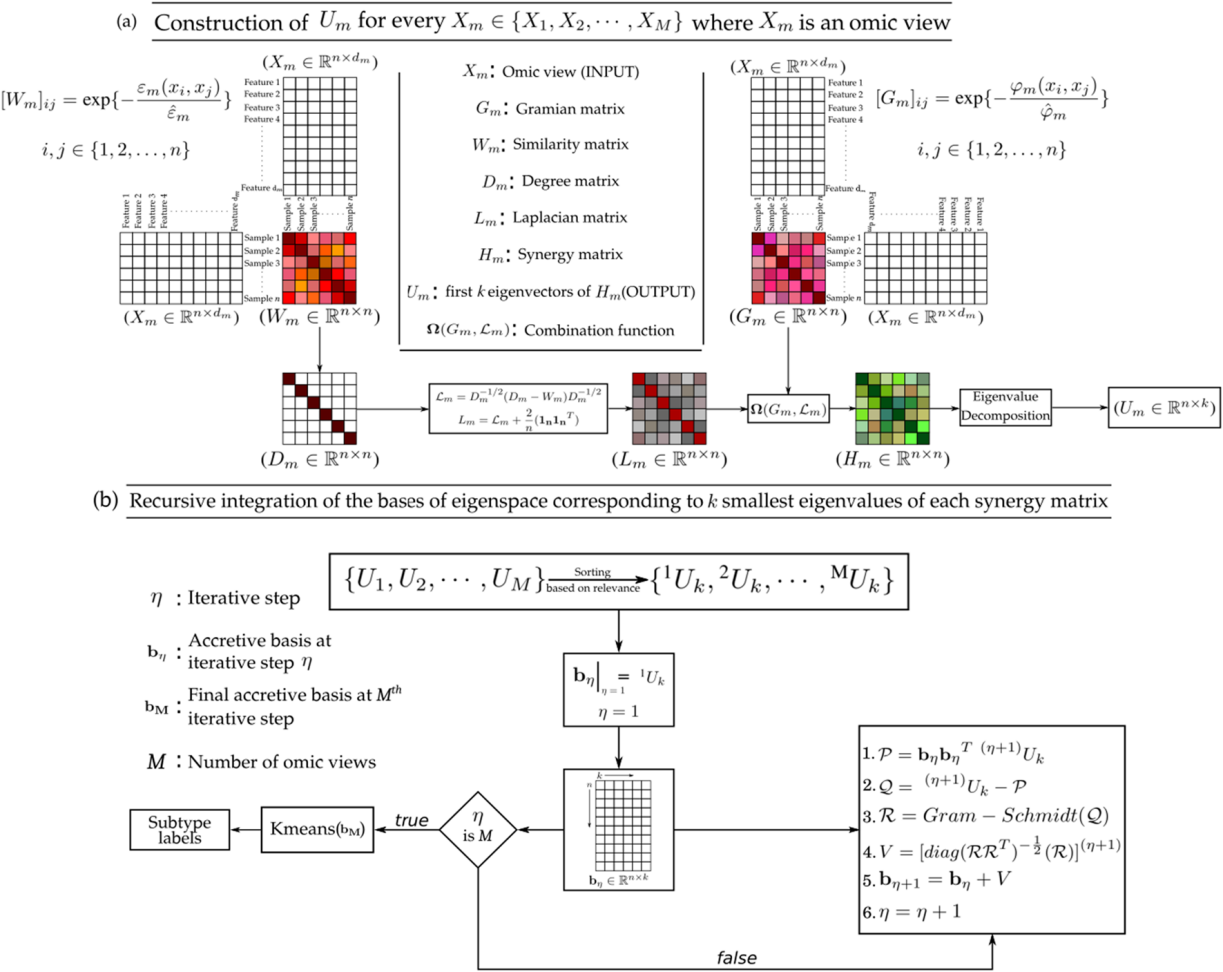
Schematic flow diagram of the proposed approach for cancer subtypes identification.

This section describes the novel algorithm designed in this study to integrate multi-omics data for cancer subtypes identification. The proposed method integrates multi-view data using a recursive multi-kernel integration function. It uses the graphical representation to harness the best picture of sample similarities from each of the omic views and explores each view’s statistical property. The schematic workflow of RISynG is presented in Fig. 1. Before moving to the steps of the proposed algorithm, first, the required analytical formulations are discussed.

### Gramian matrix and kernel trick

Gramian matrix, $G=[g_{ij}]_{n\times n}$ is a Hermitian matrix, in which each element is a pairwise Hermitian inner product of the vectors in a Hausdorff pre-Hilbert space, *V* = $\{{v_{1},v_{2},v_{3}, \ldots ,v_{n}}\}$.

$$\begin{aligned} G(v_{1},\dots ,v_{n})= \begin{bmatrix}<v_{1},v_{1}>&{} \dots &{}<v_{1},v_{n}>\\<v_{2},v_{1}> &{} \dots &{}<v_{2},v_{n}> \\ \vdots &{} \ddots &{} \vdots \\<v_{n},v_{1}> &{} \ldots &{} <v_{n},v_{n}> \\ \end{bmatrix} \quad , v_{i}\in {\mathbb {R}}^d. \end{aligned}$$

The Hermitian inner product space is accompanied by the geometric notions associated with the vectors, such as the length and the angle between two vectors. Since *G* is a Hermitian matrix, it inherits all the properties portrayed by a Hermitian matrix. A few of the relevant properties are enlisted below^28^.

#### Property 1

*All the eigenvalues of* *G* *are real*.

#### Proof

Eigenvalues of a matrix are the roots of its characteristic equation. The characteristic equation for matrix *G* is written as:

1 $$\begin{aligned} \det {(\lambda I-G)}=0. \end{aligned}$$

Let the root be some complex number $\lambda = a+ib, a,b\in {\mathbb {R}}, b\ne 0$ and *I* be the identity matrix of same order. Since, at this value of $\lambda$, the characteristic equation has a non-empty kernel, there must exist a vector $u=x+iy, x,y\in {\mathbb {R}}$ such that:

2 $$\begin{aligned} {Gu=\lambda u}, \end{aligned}$$

or,

3 $$\begin{aligned} {G(x+iy)=(a+ib)(x+iy)}. \end{aligned}$$

Taking adjoint of this equation we get,

4 $$\begin{aligned} {G(x-iy)=(a-ib)(x-iy)}. \end{aligned}$$

If $x+iy$ and $x-iy$ were two different eigenvectors of matrix *G*, then their inner product $x^2+y^2$ would have been 0 because of the mutual orthogonality among the eigenvectors. That is not possible until *x* and *y* are 0, in which case, (3) and (4) would be indifferent. That is possible only if the initial assumption is contradicted and *b* is allowed to be 0 for all eigenvectors *x*. Hence, it is proved that all the eigenvalues of *G* are real. $\square$

#### Property 2

*G* *is symmetric and positive semi-definite matrix*.

#### Proof

Pertaining to the fact that $v_{i}\in {\mathbb {R}}^d$, the following should hold for some set of vectors *x*.

5 $$\begin{aligned} {x^{\textsf {T}}{G} x=\sum _{i,j}x_{i}x_{j}\left\langle v_{i},v_{j}\right\rangle =\sum _{i,j}\left\langle x_{i}v_{i},x_{j}v_{j}\right\rangle }. \end{aligned}$$

According to the elementary property of inner products, $\square$

${\displaystyle \langle x+y,x+y\rangle =\langle x,x\rangle +\langle x,y\rangle +\langle y,x\rangle +\langle y,y\rangle \,.}$ It implies that the sum of inner products in (5) can be taken forward as,

6 $$\begin{aligned} {\left\langle \sum _{i}x_{i}v_{i}, \sum _{j}x_{j}v_{j}\right\rangle =\left\| \sum _{i}x_{i}v_{i} \right\| ^{2}\ge 0.} \end{aligned}$$

Therefore, *G* is positive semi-definite matrix.

#### Property 3

*All the eigenvalues of G are non-negative*.

#### Proof

Property 2 implies $x^{\textsf {T}}{G} x\ge 0$. Substituting the value of *Gx* from (2) into it,

7 $$\begin{aligned} {x^{\textsf {T}}{G} x=\lambda x^{\textsf {T}}x}\ge 0. \end{aligned}$$

Since $x^{\textsf {T}}x$ is positive for all eigenvectors, therefore, $\lambda \ge 0$. Hence proved.

The previously described premise is often used in various methods of dimensionality reduction. Algorithms like Principal Component Analysis and its variants utilize kernel trick to map the observations into a higher dimension to make the data linearly separable. It is equivalent to projecting the mean-centered data onto a subspace on which its variance is maximum^29^. It is shown by Bernhard Scholkopf et al.^30^ that algorithms like KPCA use a kernel function $\varvec{\kappa }$ to essentially learn a mapping function $\phi$ for the input space ${\mathbb {R}}^n$ into a high-dimensional Hilbert space $\mathbf{F}$, which can be called as feature space. The process is demonstrated in (8) and (9).

8 $$\begin{aligned} {\phi :{\mathbb {R}}^n \rightarrow \mathbf{F}}. \end{aligned}$$

Therefore, for a data point $v=(x_1,\dots ,x_n), x_i \in {\mathbb {R}}^d$, mapping into a feature space ${\mathbb {R}}^{n+k}$ will be given by

9 $$\begin{aligned} {\phi (v)=(x_1,\dots ,x_n,p_1,\dots ,p_k)\in {\mathbb {R}}^{n+k}}, \end{aligned}$$

where, the value of $p_i$ depends upon the kernel that has been used for the mapping; however, kernels do not explicitly project the data into that high dimensional feature space; rather, it generates a Gramian matrix *G* of the mapped data in the aforementioned feature space $\mathbf{F}$. Generated Gramian matrix enables the input data to be operated in that high-dimensional feature space^31^. If $X=(x_1\dots x_n), x_i\in {\mathbb {R}}^{d}$ represent the input data. The corresponding Gramian matrix is given by

10 $$\begin{aligned} {[G]_{ij}=\kappa ({x_i, x_j}) = \phi ({x_i}) \phi ({x_j})^T, {x_i},{x_j}\in X}. \end{aligned}$$

Let $G=U\Sigma U^T$ represent the eigen decomposition of *G*, where *U* is a matrix containing the eigenvectors of matrix *G*, arranged column-wise in descending order of their corresponding eigenvalues, which are present in the same fashion in the diagonal matrix $\Sigma$ as shown in (11) and (12).

11 $$\begin{aligned} U= & {} [u_1,\dots ,u_n], \end{aligned}$$

12 $$\begin{aligned} \Sigma= & {} diag(\lambda _1,\lambda _2,\dots ,\lambda _n). \end{aligned}$$

Here, $$\lambda _1\ge \dots \ge \lambda _n\ge 0$$ (see *Property 3* of Gramian matrix), $$u_i^Tu_i=1$$ for $$i\in \{1,2,\dots ,n\}$$ and $$Gu_i=\lambda _i u_i$$. Also note that in context of PCA *Principal Components* refers to the projection of the input data points onto the principal direction where the variance of the data is maximum. For PCA, the projection is given by $$y_i=U_k^Tx_i$$ for all $$i\in \{1,2,\dots ,n\}$$, where $$U_k$$ is a matrix of first *k* eigenvectors of *G*. However, in case of KPCA, the spectrum of *G* itself gives the projection of *X*^32^. Note that when $$\phi (v)=v$$, Gramian matrix transforms into covariance matrix. Generalising both, if $$U_k$$ represent *k* principal axes, the algorithm finds a basis of an optimal low-dimensional subspace where the $$ L_2$$-norm of reconstruction error is minimum^33^. That is, for a test sample *x*

13 $$\begin{aligned} {\underset{{U_k}}{\mathrm{arg}\,\mathrm{min} }}\, \Vert \phi (x)-U_kU_k^T\phi (x)\Vert ^2. \end{aligned}$$

In addition to dimensionality reduction, principal component analysis can also be used for *k*-clustering using a heuristic based *k*-means algorithm. This is done by performing *k*-means clustering in the projected space, as shown in heuristic *k*-means algorithm described in^34^.$\square$

### Graph Laplacian

Any set of observations appear to have an emergent behaviour to evince the properties of a graph when operated in a clustering pipeline. Therefore, given a set of data points $X=(x_1,x_2,\dots ,x_n) \in {\mathbb {R}}^{d\times n}$ and a notion of similarity between any two points $x_i$,$x_j\in X$, an undirected similarity graph $S=(V,E)$ can be constructed out of them such that each vertex $v_i\in V$ represent a data point $x_i$, and $(v_i,v_j)\in E$ represent the edge between vertices $v_i$ and $v_j$. With each edge, there is an associated edge weight $e_{ij}$ that represent the similarity between the corresponding data points. Let the similarity matrix be $W(i,j)=[e_{ij}]_{n\times n}$. The degree $d(v_i)$ associated with each node $v_i$ is given by

14 $$\begin{aligned} d(v_i)=|\{v_j\in V|\{v_j,v_i\}\in E\text { or }\{v_i,v_j\}\in E\}|=\sum _{j=1}^ne_{ij}. \end{aligned}$$

The degrees of all the nodes/vertices can be wrapped in matrix form as shown in (15)

15 $$\begin{aligned} D=diag(d_1,d_2,\dots ,d_n). \end{aligned}$$

These matrices act as a precursor for constructing a matrix of algebraic importance, called *Laplacian matrix*. The data can be composed as a discrete graph form by making graph Laplacian of its continuous representations like vector space or Riemannian manifolds. Laplacian matrix has many variants, so much so, that depending on the problem and available data, authors device their own version of graph Laplacian matrix^35^. The simplest graph Laplacian, is given by ($D-W$). It is called unnormalise graph Laplacian matrix. However, in the proposed algorithm, the normalised graph Laplacian matrix has been used. That is,

16 $$\begin{aligned} {\mathscr {L}}=D^{-1/2}(D-W)D^{-1/2}=I-D^{-1/2}WD^{-1/2}, \end{aligned}$$

where $D^{-1/2}=diag(d_1^{-1/2},d_2^{-1/2}, \dots ,d_n^{-1/2})$ and *I* is the identity matrix of appropriate order. Considering the fact that similarity matrix is a Gramian matrix, it is apparent that Gramian and Laplacian are not much different. Laplacian can be characterised as the Gramian normalised over the degree matrix. The distinction between unnormalised and normalised graph Laplacian is better apparent in light of spectral clustering. Consider a strongly connected graph $S=(V,E)$. The purpose of clustering is to come up with the subsets of points according to their similarity, such that the similar points lie in the same subset. It is equivalent to finding the *partitions* of a graph such that the edge between different partitions has minimum weights. For two disjoint subsets $A, B\subset V$ corresponding to two different partitions, the cut size is given by

17 $$\begin{aligned} cut(A,B)=\sum _{i\in A,j\in B}e_{ij}. \end{aligned}$$

Let there be *k* clusters in the data. The aim of clustering is to find *k* such partitions ${\mathbf{A}=(A_1,A_2,\dots ,A_k)}$, such that the size of the cuts, as shown in (17), over all the partitions is minimum. That is

18 $$\begin{aligned} \underset{A_1,\dots ,A_k}{\min }{{\text {cut}}} (A_i:0\ge i\ge k):=\sum _{i=1}^k{cut(A_i,\bar{A_i})}, \end{aligned}$$

where $\bar{A_i}$ is the complement of $A_i$. This is called the *mincut* problem. However, solving (18) alone does not achieve reliable clustering results. For example, for $k=2$, partitioning one vertex from the rest of the graph can also be a valid solution as per mincut. In clustering, each cluster needs to accommodate a reasonably large partition to be considered credible. Therefore, the objective function is redefined in following two ways

19 $$\begin{aligned} \underset{A_1,\dots ,A_k}{\min }{{\text {RatioCut}}} (A_i:1\ge i\ge k):= & {} \sum _{i=1}^{k} {\frac{cut(A_i,\bar{A_i})}{|A_i|}}, \end{aligned}$$

20 $$\begin{aligned} \underset{A_1,\dots ,A_k}{\min }{{\text {NCut}}} (A_i:1\ge i\ge k):= & {} \sum _{i=1}^{k} {\frac{cut(A_i,\bar{A_i})}{vol(A_i)}}, \end{aligned}$$

where $|A_i|$ represent the number of vertices in partition $A_i$ and $vol(A_i)=\sum _{v_j\in A_i}{d_j}$.

However, solving these minimisation problems is NP hard. Laplacian matrix is an utility that can be used to approximate these minimisation problem. Consequently, unnormalised Laplacian serves in the approximation of the minimization of RatioCut, while normalised Laplacian serves in the approximation of the minimization of NCut. Therefore, the approximated objective function using normalised Laplacian is given by (21).

21 $$\begin{aligned} \underset{U_k}{\min }{{\text {tr}}} (U_k^T{\mathscr {L}}U_k), \text { subjected to }U_k^TU_k=I. \end{aligned}$$

The above expression is minimum when $U_k\in {\mathbb {R}}^{n\times k}$ is a matrix containing eigenvectors corresponding to *k* smallest non-zero eigenvalues of matrix ${\mathscr {L}}$. This matrix is used to embed the data into a *k* dimensional euclidean space spanned by the vectors in matrix *U*, in which grouping of the data points is arguably easy even with simpler techniques like *k*-means. The described practice is known as *Laplacian embedding*. The embedded data is then subjected to *k*-means clustering algorithm for cluster discovery, as shown in Normalised Spectral Clustering presented in Ref.^36^.For a strongly connected graph with single component, the eigenvector corresponding to the trivial solution (i.e. $\lambda =0$) of the eigenvalue problem of matrix ${\mathscr {L}}$ is a column vector of n ones. Therefore, ${\mathscr {L}}{} \mathbf{1}_{n}=0$ where $\mathbf{1}_{n}=(1,\dots ,1)^T$. If the graph happens to have more than one components, then the multiplicity *k* of eigenvalue 0 if equal to the number of connected components in the graph. Nonetheless, with respect to clustering, the eigenvector(s) corresponding to eigenvalue 0 should be omitted while performing Laplacian embedding. It can be done by introducing a minor change in the matrix.

22 $$\begin{aligned} L={\mathscr {L}}+{\frac{2}{n}}{(1_n 1_n^T)}. \end{aligned}$$

If the eigenpairs of ${\mathscr {L}}$ are given by

$$\begin{aligned} {\varvec{\Gamma }}({\mathscr {L}})=\{(\lambda _1,f_1), (\lambda _2,f_2),\dots ,(\lambda _n,f_n)\} \end{aligned}$$

then, the eigenpairs of (22) are given by

$$\begin{aligned} &{\varvec{\Gamma }}(L)=  {} \{(\lambda _2,f_2),(\lambda _3,f_3),\dots ,(\lambda _1+2,f_1)\}\\&{\text { { where,} }} 0=\lambda _1<\lambda _2\dots \le \lambda _n\le 2\text { and }f_1=\mathbf{1}_n. \end{aligned}$$

Hence, the new eigenvalue problem becomes

23 $$\begin{aligned} Lv={\mathscr {L}}v+{\frac{2}{n}}(1_n1_n^T)v=\lambda v. \end{aligned}$$

By modifying the matrix to *L*, the initial *k* eigenvectors can be taken right away. This trick works because of the fact that for all the pairs in ${\varvec{\Gamma }}({\mathscr {L}})$ except $(\lambda _1,f_1)$, the matrix *L* gets reduced to ${\mathscr {L}}$. Hence, set ${\varvec{\Gamma }}(L)$ is going to have all the eigenpairs that are in ${\varvec{\Gamma }({\mathscr {L}})}$, except $(\lambda _1,f_1)$. While at $v=f_1=\mathbf{1}_{n}$,

24 $$\begin{aligned} L\mathbf{1}_{n}={\mathscr {L}}{} \mathbf{1}_{n}+{\frac{2}{n}}(\mathbf{1}_n1_n^T) \mathbf{1}_{n}=\lambda _1\mathbf{1}_{n}+2\mathbf{1}_{n}=(\lambda _1+2)\mathbf{1}_{n}. \end{aligned}$$

Therefore, in the new set $\varvec{\Gamma }(L)$, the rank of all the eigenvalues greater than $\lambda _1$ gets reduced by one and $\mathbf{1}_{n}$ becomes the eigenvector corresponding to the largest eigenvalue. Laplacian matrix has certain properties which are exploited by many clustering techniques like the one shown above. Some of the relevant properties are as following.

#### Property 1

*For every vector* $f\in {\mathbb {R}}^n$, ${\mathscr {L}}$ *satisfies the following condition*

25 $$\begin{aligned} {f^\prime {\mathscr {L}}f={\frac{1}{2}} \bigg(\sum _{i,j=1}^ne_{ij} \bigg({\frac{f_i}{\sqrt{d_i}}}-{\frac{f_j}{\sqrt{d_j}}\bigg)^2\bigg)}} \end{aligned}$$

#### Proof

By the definition of degree, $d_i=\sum _{j=1}^ne_{ij}$. Therefore,

$$\begin{aligned}&  f^{\prime }{\mathscr {L}f  = f^{\prime }(I-D^{-1/2}WD^{-1/2})f} \\& \quad =\sum _{i=1}^nf_i^2-\sum _{i,j=1}^n{\frac{f_i}{\sqrt{d_i}}}{\frac{f_j}{\sqrt{d_j}}e_{ij}} \\& \quad  ={\frac{1}{2}}\left( \sum _{i=1}^n{\frac{f_i^2}{d_i}}d_i+\sum _{j=1}^n{\frac{f_j^2}{d_j}}d_j-2\sum _{i,j=1}^n{\frac{f_i}{\sqrt{d_i}}}{\frac{f_j}{\sqrt{d_j}}}e_{ij}\right) \\&  \quad ={\frac{1}{2}}\left( \sum _{i,j=1}^n{\frac{f_i^2}{d_i}}e_{ij}+{\frac{f_j^2}{d_j}}e_{ij}-2{\frac{f_i}{\sqrt{d_i}}}{\frac{f_j}{\sqrt{d_j}}}e_{ij}\right) \\& \quad  ={\frac{1}{2}}\left( \sum _{i,j=1}^ne_{ij}\left( {\frac{f_i}{\sqrt{d_i}}}-{\frac{f_j}{\sqrt{d_j}}}\right) ^2\right) . \end{aligned}$$

Hence proved.$$\square $$

#### Property 2

${\mathscr {L}}$ *is symmetric and positive semi-definite matrix*.

#### Proof

From (16), the symmetry of the matrix is fairly evident. Also, from the property 1, ${f^\prime {\mathscr {L}}f}\ge 0$ for all $f\in {\mathbb {R}}^n$. Hence, it is provrd that ${\mathscr {L}}$ is symmetric and positive semi-definite matrix. $\square$

#### Property 3

*All eigenvalues of* ${\mathscr {L}}$ *are non-negative*.

#### Proof

Property 1 implies ${f^\prime {\mathscr {L}}f}\ge 0$. Substituting ${\mathscr {L}}f=\lambda f$, we get ${{f^\prime {\mathscr {L}}f}=\lambda x^{\textsf {T}}x}\ge 0$. Since $f^\prime f$ is positive for all eigenvectors, therefore, $\lambda \ge 0$. Hence proved.$\square$

### RISynG algorithm

For grouping the cancer patients into clusters, each omic view is represented as a graph using two representation matrices, that is the Gramian matrix and the Laplacian matrix. Each of the representation matrices attributes the similarity network of the samples with a notion of similarity between the samples. Consider a view $X_m=(x_1,x_2,\dots ,x_n)$, $x_i\in {\mathbb {R}}^{d_m}$ corresponding to *m*th omic-source. If $\rho (x_i,x_j)$ denotes the distance between $x_i$ and $x_j$ $\in X_m$, then the similarity $w(x_i,x_j)$ between them is given by:

26 $$\begin{aligned} w(x_i,x_j)=\text {exp} \left\{ -{\frac{\rho (x_i,x_j)}{\sigma }}\right\} , \end{aligned}$$

where $\sigma$ is a free parameter adjusted as per the intrinsic properties of the data when subjected to clustering model. For the cancer data used in this study, the $\sigma$ is given by $\sigma ={\text {max}(\frac{\rho (x_i,x_j))}{2}}$ for all $x_i,x_j\in X_m$. It has been assumed in the proposed method that multi-views may constitute different cluster manifolds when learnt on a particular similarity measure. Therefore, predicted clusters would be apparent, and in strong concordance with the clinical clusters if pairwise sample similarity is computed in data-dependent multi-kernel approach. It was found that in some views correlation distance was prominently reflecting cluster manifold that concurred with the natural clusters, while some of them showed proclivity towards Euclidean distance, and the rest seemed to accommodate parts of both. All things considered, two different graph representation matrices have been formulated, Gramian matrix and Laplacian matrix, both with different measures of similarity. Let for $X_m$, the correlation distance between $x_i$ and $x_j$ be given by $\varphi _m(x_i,x_j)$ and the squared Euclidean distance be given by $\varepsilon _m(x_i,x_j)$. If $\hat{\varphi }_m$ and $\hat{\varepsilon }_m$ denotes the maximum pairwise correlation distance and squared Euclidean distance respectively, then Gramian matrix $G_m$ and similarity matrix $W_m$ are given by

27 $$\begin{aligned}{}[G_m]_{ij}= & {} w_G(x_i,x_j)=\text {exp} \left \{-{\frac{\varphi _m(x_i,x_j)}{\hat{\varphi }_m}} \right \} \left \{-{\frac{\varphi _m(x_i,x_j)}{\hat{\varphi }_m}} \right \} ,  \quad where \;  i,j\in \{1,2,\dots ,n\}, \end{aligned}$$

28 $$\begin{aligned} _{ij}= & {} w_L(x_i,x_j)=\text {exp} \left \{-{\frac{\varepsilon _m(x_i,x_j)}{\hat{\varepsilon }_m}} \right \} ,  \quad where \; i,j\in \{1,2,\dots ,n\}. \end{aligned}$$

The matrix articulated in (28) is a crucial precursor for the construction of Laplacian matrix. Laplacian matrix is constructed by normalising $W_m$ by the degree matrix $D_m$ of its associated graph as in Eqs. (15) and (16). Hence, required representation matrices for each view $X_m$, $m\in \{1,2,\dots ,M\}$ are given by (27) and (29).

29 $$\begin{aligned} {\mathscr {L}}_m=D_m^{-1/2}(D_m-W_m)D_m^{-1/2}=I-D_m^{-1/2}W_mD_m^{-1/2}. \end{aligned}$$

So obtained laplacian matrix is then modified as described in Eq. (22)

30 $$\begin{aligned} L_m={\mathscr {L}}_m+{\frac{2}{n}}(1_n 1_n^T). \end{aligned}$$

It is apparent from the discussion presented under the heading Gramian Matrix and Kernel Trick and Graph laplacian that the matrix $U_k$ from Gramian matrix has the same role as that from Laplacian matrix. Therefore, for combining the information encoded in these matrices, a parameterised combination function ${\varvec{\Omega }}(\cdot ,\cdot )$ can be used, hence obtaining a synergy matrix of representation matrices. If $G_m$ is the Gramian matrix and $L_m$ is the Laplacian matrix of omic-view $X_m$, then the synergy matrix is given by:

31 $$\begin{aligned} {\varvec{\Omega }}(G_m,{\mathscr {L}}_m) = H_m =\beta G+(1-\beta )L,  \quad where  \; 0\le \beta \le 1. \end{aligned}$$

Consequently, the corresponding objective functions, (13) and (21) also combines to optimise over $U_k\in {\mathbb {R}}^{n\times k}$.

32 $$\begin{aligned} \begin{aligned} \underset{U_k}{\min }{\beta \Vert X-U_kU_k^TX\Vert _F+ (1-\beta ){\mathbf{tr}}}(U_k^T{\mathscr {L}}U_k) ,  \text { subjected to }U_k^TU_k=I. \end{aligned} \end{aligned}$$

Some of the relevant properties of synergy matrix $H_m$ are:

#### Property 1

$H_m$ *is symmetric and positive semi-definite matrix*.

#### Proof

$H_m$ can be called a positive semi-definite matrix if and only if $v^TH_mv\ge 0$ for all $v\in {\mathbb {R}}^n$. Also, from the properties of the Graph Laplacian and the Gramian, it is evident that both *L* and *G* satisfies this condition. Therefore,

33 $$\begin{aligned} v^TH_mv=\beta v^TGv+(1-\beta )v^TLv\ge 0. \end{aligned}$$

In addition to that, since $H_m$ is a summation of symmetric matrices, it is also symmetric. Hence, it is proved that $H_m$ is a symmetric and positive semi-definite matrix.$\square$

Given *Property 1*, rest of the properties are its direct consequence.

#### Property 2

All the eigenvalues of $H_m$ are real.

#### Property 3

*All the eigenvalues of* $H_m$ *are non-negative*.

#### Recursive multi-kernel integration

After generating synergy matrices for all the views of the dataset, the next step is to integrate the information obtained from each of them. However, before moving to the integration step, the proposed approach needs these matrices to be arranged based on their relative relevance for cluster discovery. It is apparent that the better views would encode the cluster structure better. As a consequence of that, they would depict better cluster validity indices as well. Therefore, the sorting of synergy matrices have been done based on cluster validity indices such as silhouette index. Suppose $\mathbf{H}=\{H_1,\dots , H_M\}$ be the set of synergy matrices of a dataset with *M* views. Let the sorted set be $\mathbf{H}^{\prime }=\{^1H, \dots , ^MH\}$, where the superscript *i* denotes the relevance of the corresponding synergy matrix $^iH$, $^1H$ being the most relevant. Additionally, let every $^iU_k$ from the set $\mathbf{U}=\{^1U_k, \dots , ^MU_k\}$ represent the basis of eigenspace corresponding to *k* smallest eigenvalues of matrix $^iH$.

Next, a method for combination has been proposed which distills the cluster information from each of the synergy matrix one by one, in an iterative fashion. While doing that, it subtly takes care of enriching the information coming from the relevant matrices. The way that the synergy matrices has been made, it is apparent that it is their basis of the eigenspace that brings out the latent cluster structure in the corresponding view. Therefore, the proposed method uses a recursive function to exploit this fact for integration as well as enrichment of the relevant views of the dataset. The recursive formula can be written as:

34 $$\begin{aligned} \begin{aligned} \mathbf{k}_{\eta +1}:=\mathbf{k}_{\eta }\otimes {{\mathscr {N}}}(\mathbf{k}_{\eta },^{(\eta +1)}U) ,  \text {where }  \mathbf{k}_1=^1H \text { and }\eta =1,\dots ,M. \end{aligned} \end{aligned}$$

Here $\mathbf{k}_{\eta }$ is called accretive matrix of $\eta$th recursive step. Non-cumulative operator $\otimes$ signifies the integration operation. That is, for $A\in {\mathbb {R}}^{n\times n}$ and $U\in {\mathbb {R}}^{n\times k}$, where *A* has its *k* smallest eigenvectors in $V\in {\mathbb {R}}^{n\times k}$, and *U* is a basis matrix, the expression $A\otimes U$ evaluates to an accretive matrix $A^\prime \in {\mathbb {R}}^{n\times n}$ with *k* smallest eigenvectors given by $V+U$. Other eigenvectors of *A* are irrelevant for this discussion. Let the basis of eigenspace of $A^\prime$ be known as accretive basis and associated subspace as accretive subspace. Also, let the accretive basis corresponding to *k* smallest eigenvectors of $\mathbf{k}_{\eta }$ be given by $\mathbf{b}_{\eta }$.

In extension to that, for enriching relatively relevant views, the proposed method uses an orthogonalising-normalising function ${{\mathscr {N}}}(\cdot ,\cdot )$. To ensure the accumulation of only the essential cluster information, the proposed approach acquires the basis of that projection of synergy matrix eigenspace which is orthogonal to the accretive subspace at that recursive step. The idea is similar to eigenspace updation for integrative clustering as performed in Ref.^18^. This function does not normalise the synergy matrix per se, rather, it normalises the basis of the described projection subspace. The computation starts by instantiating $\mathbf{k}_{1}=\text { }^1H$ so that $\mathbf{b}_{\eta }$ becomes $^1U_k$. Therefore, at ($\eta +1$)th recursive step ($\eta \in \{0,1,\dots ,M\}$), one should have accretive matrix $\mathbf{k}_{\eta }$ and eigenspace basis $^{(\eta +1)}U_k$ of synergy matrix $^{(\eta +1)}H$. Subsequently, processing within orthogonalising-normalising function ${{\mathscr {N}}}(\mathbf{k}_{\eta },^{(\eta +1)}U_k)$ renders the final basis matrix in four steps:

First, computing the basis ${\mathscr {P}}$ of the projection subspace, which is given by:

35 $$\begin{aligned} {\mathscr {P}}=\mathbf{b}_{\eta }{} \mathbf{b}_{\eta }^T\text { }^{(\eta +1)}U_k. \end{aligned}$$

Second, computing the residual component of the synergy matrix eigenspace ${\mathscr {Q}}$ which is given by subtracting the above-mentioned projected component from $^{(\eta +1)}U_k$ as:

36 $$\begin{aligned} {\mathscr {Q}}=\text { }^{(\eta +1)}U_k-{\mathscr {P}}. \end{aligned}$$

In the third step, ${\mathscr {Q}}$ is subjected to Gram-Schmidt orthogonalisation to yield the final basis ${\mathscr {R}}$. This basis cannot be integrated with the eigenspace of accretive matrix, therefore it needs to be normalised on the basis of its relevance. So, the fourth step of normalization is performed as:

37 $$\begin{aligned} {{\mathscr {N}}}(\mathbf{k}_{\eta },^{(\eta +1)}U_k)=V, \quad  where \;  V=[diag({\mathscr {R}} {\mathscr {R}}^T)^{-{\frac{1}{2}}}({\mathscr {R}})]^{(\eta +1)} \end{aligned}$$

Here the notation $[\cdot ]$ denotes that the subsequent operations are done in element-wise fashion. The resultant *V* matrix is called as orthogonalised-normalised basis matrix. After the end of the process, the final accretive matrix $\mathbf{k}_{M}$ is obtained whose first *k* eigenvectors in the matrix $\mathbf{b}_{M}\in {\mathbb {R}}^{n\times k}$ holds the cluster structure. Hence, performing *k*-means on the rows of the matrix $\mathbf{b}_{M}$ returns the cluster labels for each sample. The proposed algorithm is described in Algorithm 1.
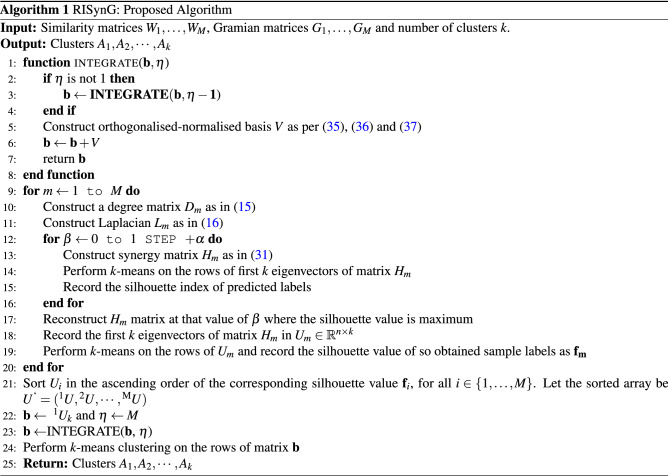


### Computational complexity

For the proposed algorithm, given *M* similarity matrices and Gramian matrices with *n* samples under study, the computation starts with constructing degree matrix $D_m$ for each of the *M* views. The complexity of this step is bounded by $O(n^2)$ for each view. In the next step, the Laplacian matrix is made with a complexity of $O(n^3)$. Let the number of iterations (regulated through parameter $\beta$) to learn the synergy matrix’s best composition in steps 12 to 16 be $t_\beta$. However, it has been found that for the datasets used in this study, the value of $t_{\beta }=10$ suffices. Iterating $\beta$ from 0 to 1 with an increment of 0.1 with each iteration can produce an optimal combination ratio for the representation matrices. However, here, the increment step has been referred to as $\alpha$ for consistency. Assuming $t_{max}$ be the highest iteration by the *k*-means clustering algorithm the complexity of the aforesaid steps becomes $O(t_{\beta }n^3+t_{\beta }t_{max}nk^2+t_{\beta }n)$. Where $t_{\beta }n^3$ comes from the complexity of eigenvalue decomposition of synergy matrix, $t_{\beta }t_{max}nk^2$ is for the step where *k*-means clustering is performed, and $t_{\beta }n$ is for the *f*-measure calculation. Therefore, the complexity of steps formulated from 12 to 16 turns out to be bounded by $O(t_{\beta }n^3)$. Steps 17 to 19 are doing the same processing as previously, just at the optimal value of $\beta$. Hence, they are also bounded by $O(t_{\beta }n^3)$. Summing up all the steps from 9 to 20 for *M* views, the complexity of $O(Mn^2+Mn^3+Mt_{\beta }n^3)$ reduces to $O(Mt_{\beta }n^3)$. Sorting can be done at *O*(*MlogM*). After that, an accretive basis is constructed as defined in the function INTEGRATE($\mathbf{b},\eta$). Step 5 consists of the construction of ${\mathscr {P}}$, ${\mathscr {Q}}$ and orthogonalized-normalized matrix *V*. In this step, two matrix multiplication operations are bounded under the complexity of $O(n^2k)$. Gram-Schmidt orthogonalization and normalization step combined has a complexity of $O(n^2)$. Therefore, step 5 has a complexity of $O(n^2k)$. Step 6 is matrix addition with complexity *O*(*nk*), but step 5 seem to dominate over that. In addition to that, since the function runs $(M-1)$ times, the complexity from steps 21 to 23 becomes $O(MlogM+Mn^2k)=O(Mn^2k)$. After the construction of the accretive basis, *k*-means is performed, which, as explained previously, has time complexity $O(t_{max}nk^2)$. Considering everything, the overall complexity of RISynG comes out to be $O(Mt_{\beta }n^3+Mn^2k+t_{max}nk^2) = O(Mt_{\beta }n^3)$.

### Significance of proposed algorithm

There are some aspects of the proposed algorithm that enhance its performance and make it unique from the other algorithms designed to identify cancer subtypes. Although each omic-view in the cancer dataset has its distinct cluster structure, the knowledge of cancer biology suggests that no omics-source to which each view belongs can dictate the final cancer subtype alone. Instead, all the omics sources collectively manifest the cancer subtype in a sample. Therefore, multi-view integration is critical to a sensible and clinically relevant clustering. The proposed approach can be broken down into three operative steps: (1) construction of representation matrices for each view, (2) construction of synergy matrix for each view, and (3) construction of accretive basis through recursive multi-kernel integration of synergy matrices. These steps make the proposed algorithm more effective in the following manner:

1. *Construction of representation matrices* To group the cancer patients into clusters, each omic-view first has to be represented as similarity graphs. These similarity graphs can be interpreted through various representation matrices like the Gramian, Laplacian, and Adjacency. Each representation matrix attributes the samples’ similarity network with a notion of similarity between the samples. The proposed method assumes that multiple information sources may constitute different cluster manifolds when learned on a particular similarity measure. Therefore, predicted clusters would be apparent and in strong concordance with the clinical clusters if pairwise sample similarity is computed in a data-dependent multi-kernel approach^37^. In some views, Correlation distance was prominently reflecting cluster manifold that concurred with the natural clusters. Whereas some of them showed proclivity towards Euclidean distance, the rest seemed to accommodate both. All things considered, two different graph representation matrices have been formulated, the Gramian matrix and Laplacian matrix, both with different measures of similarity.
2. *Construction of synergy matrices* Representation matrices so constructed have two noteworthy aspects: (1) $G_m$ represents a similarity graph formed using correlation-based distance. In the correlation-based distance, two objects are considered similar if the trends among their elements are highly correlated. That means the correlation distance between two perfectly correlated samples will be 0, even though they are far apart in the euclidean space of their dimension. It is instinctive to assume the omics data to behave like that. (2) Laplacian, on the other hand, preserves the intrinsic manifold structure in the data casted on a low embedding space. To integrate these representation matrices, a combination function has been devised that takes a convex combination of both the matrices. This method of combining matrices rectifies any bias created by the dissimilarity in distance measurement used while constructing the similarity graphs. The combination function defined in (31) utilises the parameter $\beta \in [0,1]$ to capture graphs constituted by the Gramian and Laplacian. Parameter $\beta$ can only take a positive value, making the combination a convex combination of representation matrices. This parameter’s optimal value is learnt by iterating it from 0 to 1 at some incremental step size $\alpha \in (0,1)$. The datasets used in this study tend to pick up the optimal value of $\beta$ at a step size of $\alpha =0.1$. It is crucial to choose the incremental step size wisely as the number of iterations $t_{\beta }$ is directly proportional to the algorithm’s time complexity. Because the synergy matrix will ultimately affect the cluster assignment, the best way to evaluate the appropriate value of $\beta$ is to perform a provisional cluster validity test on the synergy matrix constructed with that $\beta$ using a cluster validity index like silhouette index. Algorithm-1, steps 15 to 19 formulate the described provisional cluster validity test using silhouette as a criterion.
3. *Construction of accretive basis* After the similarity between the cancer patients is captured in a refined form with the help of synergy matrices, the next step is to integrate them. Property 1 of the synergy matrix proves that $H_m$ is a positive semi-definite matrix. That makes the integration of synergy matrices a multi-kernel integration. The proposed algorithm does that by recursive multi-kernel integration by iteratively integrating each of the synergy matrices’ relevant subspace. Here, relevant subspace refers to that subspace of the matrix that purely encodes the cluster information, which in the case of synergy matrix is its eigenspace corresponding to *k* eigenvalues. Finally, an accretive basis matrix is generated. This accretive matrix is required to have more cluster information coming from relevant views. Therefore, the orthogonalizing-normalizing function is made such that the accretive basis at each recursive step gets less influenced by the irrelevant matrix.

## Description of datasets

**Figure 2:**
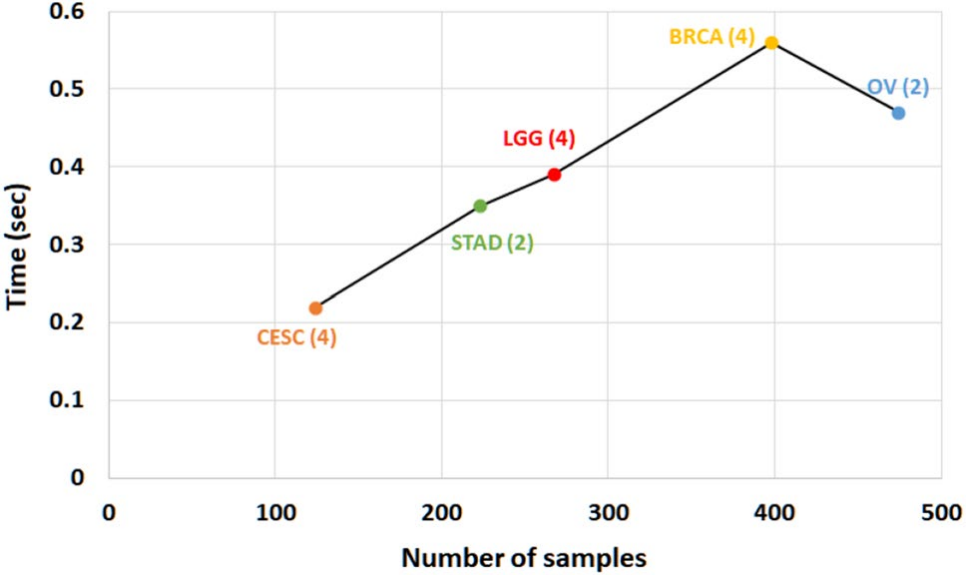
Effect of sample size and number of omic-views on the runtime of the proposed algorithm. Values in the parentheses indicate the number of omic-views.

For analysing the efficiency of the proposed algorithm for identifying cancer subtypes, it is applied to five cancer datasets taken from TCGA (https://cancergenome.nih.gov/). The datasets used are Cervical cancer (CESC), Breast cancer (BRCA), Ovarian cancer (OV), Lower-grade glioma (LGG), and Stomach cancer (STAD). Different studies have identified 4 clinically important subtypes for BRCA^9^ and STAD^38^, 3 for CESC^39^ and LGG^40^ and 2 for OV^41^. The cancer genome is neither simple nor independent but is complicated and dysregulated by multiple levels in the biological system through genomic, epigenomic, transcriptomic, proteomic levels^42^. miRNA, as one of the important regulators of gene expression, can be integrated with gene expression to identify the selective inhibition of translation or selective degradation^43–45^. Furthermore, in terms of epigenetic regulation, histone modification or DNA methylation can serve to regulate gene expression in cancer^46,47^. Also, protein expression data can be utilized for the diagnostic prognosis of cancer patients^48^. Therefore, four omic views, namely, gene expression (mRNA), microRNA expression (miRNA), DNA methylation (metDNA), and reverse-phase protein assays (RPPA), are utilized for CESC, BRCA, and LGG datasets. For STAD and OV datasets, mRNA and miRNA expression are only considered because metDNA and RPPA information are not available for most samples. To avoid involving features with too many missing values, more than 5% of missing values in all of the omic views are removed, and the rest of the missing values are replaced with 0. Sequence-based expression data are log-transformed to make the data more or less normally distributed^49^. Therefore the 0 entries from miRNA and mRNA expression data are replaced with 1 and then log-transformed with base 10. For metDNA datasets, beta values are considered. At last, variance filtering is applied to mRNA and metDNA omic views for all cancer datasets, and 2000 most variable genes and CpG locations were only considered. Table 1 contains a description of the final processed data used for this study. The datasets selected for benchmarking cover a wide range of sample sizes from 124 in CESC to 474 in OV datasets. TCGA contains several platforms for individual data types, the platforms having the largest number of matching samples across the omics are selected in the present study. The proposed algorithm can be applied to other large-scale multi-omics datasets if available; the run time will increase with the increase in sample size or the number of omic views, as shown in Fig. 2. With the increase in sample size from 124 to 474, the runtime increases from 0.22 to 0.47 s. Even though the BRCA dataset has lesser samples (398) than the OV dataset (474), the runtime for BRCA (0.56 s) is more than OV (0.47 s) because of the number of omic-views involved, which is 4 for BRCA and 2 for OV.

**Table 1:** Datasets description.

Number of features						
Datasets	Number of samples	mRNA	miRNA	metDNA	RPPA	Number of clusters
CESC	124	2000	311	2000	219	3
BRCA	398	2000	278	2000	212	4
OV	474	2000	591	–	–	2
LGG	267	2000	333	2000	209	3
STAD	223	2000	524	–	–	4

## Experimental results and discussion

The performance of the proposed approach is compared with eleven other algorithms available for cancer subtype identification. Both two-stage clustering approaches and integrative clustering approaches are considered for method comparison. The methods used for comparison are Similarity Network Fusion (SNF)^13^, Weighted Multi-View Low Rank Representation (WMLRR)^50^, Consensus Clustering (CC)^6,51^, Multi-view clustering approach with enhanced consensus (ECMC)^52^, SNF.CC (SNF merged with CC)^53^, Cluster of Cluster Assignment (COCA)^9,54^, Consensus Non-negative Matrix Factorization (CNMF)^55^, Selective Update of Relevant Eigenspaces (SURE)^18^, Convex-combination of Approximate Laplacians (CoALa)^19^, iCluster^14^, and Multi-manifold Integrative Clustering (MiMIC)^56^.

### Performance analysis on multi-omics cancer datasets

The proposed approach and the above-described methods are applied to five cancer datasets, namely CESC, BRCA, OV, LGG, and STAD, taken from TCGA. The sample clusters identified by these methods are evaluated based on several internal and external cluster evaluation indices. The cancer subtypes identified by these methods are also evaluated for their biological relevance. Next, the detailed comparative analysis of the proposed algorithm is discussed.

#### Cluster evaluation

The clusters (cancer subtypes) generated by all the methods are evaluated based on several internal and external cluster evaluation indices. These indices help get the idea of how well a method can group the samples into homogeneous clusters. Samples belonging to the same cluster should have higher similarity representing a cancer subtype, whereas samples belonging to different clusters should be highly dissimilar. How well an algorithm can capture the natural grouping present in the data can be quantified with internal validity indices. Following four internal evaluation indices are calculated in this study. Table 3, presents the internal evaluation indices for every method.

1. Silhouette Index: It measures the consistency present in the clusters. The value lies in the range $[-1,1]$. A value nearer to + 1 indicates a higher distance between the clusters, a value of 0 indicates that the sample is very close boundary between two neighboring clusters, and a negative value indicates misclassification^57^.
  38 $$\begin{aligned} {\mathbb {S}}_c = \frac{1}{c} \sum _{k=1}^{c}S(\Upsilon _k), \end{aligned}$$
  where, $$S(\Upsilon _k)$$ represents silhouette width of the obtained clusters, $$\Upsilon _k (k=1, \ldots ,c)$$ which is calculated as: $$S(\Upsilon _k)=\frac{1}{n_k}\sum _{x_i\in \Upsilon _k}^{}s(x_i)$$ where, $$n_k$$ is cardinality of $$\Upsilon _k$$ and $$s(x_i)$$ is silhouette width of sample $$x_i$$. For every sample, the silhouette width $$s(x_i)$$ is estimated as: $$s(x_i)=\frac{b(i)-a(i)}{max\{a(i),b(i)\}}$$ Here, $$a(i) = $$ average dissimilarity of $$i_{th}$$ object to all other objects in the same cluster and $$b(i) = $$ average dissimilarity of $$i_{th}$$ object with all objects in the closest cluster.
2. Dunn Index: A higher value represents better clustering solution^58^. It is defined as:
  39 $$\begin{aligned} DI = \underset{1\le i \le c}{{\text {min}}} \Big \{ \underset{1\le i \le c}{{\text {min}}} \Big \{ {\frac{\delta (C_i,C_j)}{\underset{1\le k \le c}{{\text {max}}} \small \{\Delta (C_k)\}}} \Big \}\Big \} \end{aligned}$$
  Here, $\delta (C_i,C_j) =$ distance between cluster $C_i$ and $C_j$ and $\Delta (C_k) =$ intra-cluster distance within cluster $C_k$.
3. Davies–Bouldin Index: It is defined as the ratio of within cluster dispersion to between cluster dispersion^59^. A lower value indicates better clustering.
  40 $$\begin{aligned} DB = \frac{1}{C} \sum _{i=1}^{C} (D_i) \end{aligned}$$
  Here, $D_{i}  = \max _{{j \ne i}} R_{{i,j}}$ and $R_{i,j} = \frac{S_i+S_j}{M_{ij}}$. $M_{i,j}$ is the separation between the *i*th and the *j*th cluster. $S_i$ and $S_j$ are the within cluster scatter for cluster *i* and *j* and *C* is the number of clusters.
4. Xie–Beni Index: The index for crisp clustering is estimated as:
  41 $$\begin{aligned} \text {Xie}-{\text {Beni}} = \frac{1}{N} \frac{WGSS}{ {\text{min}}_{{k < \mathop k\limits^{{\prime }} }}  \acute{\delta } (C_k,C_{\acute{k}})^2} \end{aligned}$$
  Here, $\frac{1}{N} {WGSS}$ represents the averaged-squared distance of all the points with respect to the barycenter of the cluster they belong to, and $\acute{\delta }$ a measure of the between-cluster distance^60^.

The class distribution of the cancer datasets used in this study is presented in Table 2. Except for the CESC dataset, all the other cancers have an imbalanced class. When clustering is applied to these datasets, there are chances that most of the samples get clustered into one group leading to good values for internal indices. Still, in reality, the clustering is not efficient. If the ground truth is available, the partitions created in such imbalanced data can be efficiently evaluated with external evaluation indices. In this study, five external evaluation indices are calculated to compare the clustering efficiency of the different algorithms. Considering a set of *n* objects ${{\mathbb {X}}}=\{{{\mathscr {X}}}_1, {{\mathscr {X}}}_2, \ldots ,{{\mathscr {X}}}_n\}$, suppose ${{\mathbb {C}}}=\{{{\mathscr {C}}}_1, {{\mathscr {C}}}_2, \ldots ,{{\mathscr {C}}}_R\}$ represents a partition of ${{\mathbb {X}}}$ obtained by a clustering algorithm and ${{\mathbb {K}}}=\{{{\mathscr {K}}}_1, {{\mathscr {K}}}_2,\ldots ,{{\mathscr {K}}}_C\}$ represents the ground truth or the class information. A contingency table is created to look for the overlap between the clustering result and the ground truth, where $n_{ij}=|{{\mathbb {C}}}_{i}\cap {{\mathbb {K}}}_{j}|$ is the common elements in cluster ${{\mathbb {C}}}_{i}$ and class ${{\mathbb {K}}}_{j}$. $n_i$ is the number of elements in ${{\mathbb {C}}}_{i}$ and $n_{j}$ is the number of elements in ${{\mathbb {K}}}_{j}$. The external indices are defined as:

1. F-measure (FM): The idea of precision and recall from information retrieval is merged to obtain FM. It disregards the unmatched portions of the clusters. It can attain values ranging between 0 and 1. A value nearer to 1 represents better clustering^61^.
  42 $$\begin{aligned} FM = \sum _{j=1}^{C} \frac{n_j}{n} \, \underset{i=1 \cdot \cdot \cdot R}{{\text {max}}}\, \left[ \frac{2 \times \frac{n_{ij}}{n_i} \times \frac{n_{ij}}{n_j}}{\frac{n_{ij}}{n_i}+\frac{n_{ij}}{n_j}}\right] \end{aligned}$$
2. Adjusted Rand Index (ARI): A commonly used variations of the Rand index, and takes into account agreements arising by chance given a hypergeometric distribution. In the case of ARI, the lower bound, $-k$, depends on the exact data partitioning^62^. Closer the value of ARI to 1, better is the clustering.
  43 $$\begin{aligned} ARI = \frac{\sum _{i=1}^{R} \sum _{j=1}^{C} \left( \begin{array}{c} n_{ij} \\ 2 \end{array}\right) - {\left( \begin{array}{c} n \\ 2 \end{array} \right) }^ {-1} \sum _{i=1}^{R} \left( \begin{array}{c} n_{i} \\ 2 \end{array} \right) \sum _{j=1}^{C} \left( \begin{array}{c} n_{j} \\ 2 \end{array} \right) }{\frac{1}{2} \left[ \sum _{i=1}^{R} \left( \begin{array}{c} n_{i} \\ 2 \end{array}\right) + \sum _{j=1}^{C} \left( \begin{array}{c} n_{j} \\ 2 \end{array} \right) \right] - \left( \begin{array}{c} n \\ 2 \end{array} \right) ^{-1} \sum _{i=1}^{R} \left( \begin{array}{c} n_{i} \\ 2 \end{array}\right) \sum _{j=1}^{C} \left( \begin{array}{c} n_{j} \\ 2 \end{array} \right) } \end{aligned}$$
3. Normalized Mutual Information (NMI): The inter-dependencies between cluster number and cluster quality can be quantified by NMI. It is estimated as:
  44 $$\begin{aligned} NMI({\mathbb {C}},{\mathbb {K}})=\frac{{\mathscr {I}}({\mathbb {C}},{\mathbb {K}})}{[{\mathscr {H}}({\mathbb {C}})+{\mathscr {H}}({\mathbb {K}})]/2} \end{aligned}$$
  Here, ${\mathscr {I}}$ is mutual information and ${\mathscr {H}}$ is entropy. The value ranges from 0 to 1, value nearer to 1 means better clustering^63^.
4. Jaccard Index: It is used to measure the similarity between two sets, that are clustering solution, and the class information. It is defined as:
  45 $$\begin{aligned} J({\mathbb {C}},{\mathbb {K}})= \frac{|{\mathbb {C}} \cap {\mathbb {K}}|}{|{\mathbb {C}} \cup {\mathbb {K}}|} \end{aligned}$$
  Higher the value of this index better in the clustering.
5. Purity: For estimating Purity, the clusters are first allocated to that class which is present most frequently in the cluster. Later, the accuracy of this cluster-class allocation is obtained by dividing the number of correctly assigned objects to total number of objects^63^. The equation for calculating Purity is:
  46 $$\begin{aligned} Purity({\mathbb {C}},{\mathbb {K}})=\frac{1}{n}\sum _{i}max_{j}|C_i \cap K_j| \end{aligned}$$
  Purity ranges from 0 to 1, a value closer to 1, better is the clustering.

Based on these five external evaluation indices, it is observed that the proposed algorithm outperforms in CESC, BRCA, LGG, and STAD datasets. OV cancer is the only case where the proposed approach cannot work that well. Suppose all the datasets are considered together to rank the clustering efficiency of all the algorithms under study, considering all the external indices. In that case, the proposed method stands first by attaining a maximum value for 20 times out of 25. The execution times reported in Table 3 show that RISynG is faster than other algorithms.

**Table 2:** Cancer subtypes description: actual class distribution.

Datasets	Subtypes	Number of samples
CESC	Keratin low squamous	37
	Keratin high squamous	58
	Adenocarcinoma	29
	Luminal A	80
BRCA	Luminal B	49
	Her-2 enriched	171
	Triple negative/basal like	98
OV	Neoplasm histological grade 3	417
	Neoplasm histological grade 2	57
LGG	IDH mutation without 1p/19q codeletion	134
	IDH mutation with 1p/19q codeletion	84
	Wild type IDH subtype	49
	Microsatelite instability (MSI)	45
STAD	Epstein–Barr virus (EBV)	17
	Chromosomal instability (CIN)	111
	Genomically stable (GS)	50

**Table 3:** Comparative cluster analysis of proposed and existing approaches.

Datasets	Methods	Internal evaluation indices				External evaluation indices					Time (s)
		Silhouette	Dunn	DB	Xie-Beni	F-measure	ARI	NMI	Jaccard	Purity	
CESC	SNF	0.4009138	0.4454051	1.2271228	1.0368490	0.7258065	0.4066304	0.4872207	0.4482759	0.7258065	0.39
	CC	0.6558044	0.2434998	0.3457636	0.8466638	0.6774194	0.3682621	0.3919370	0.4441509	0.6774194	31.61
	CNMF	**0.8849808**	**0.5815151**	**0.1346099**	**0.1404269**	0.6693548	0.4280599	0.3887215	0.4761179	0.6693548	65.7
	ECMC	–	–	–	–	0.5943548	0.4296452	0.4684325	0.4978563	0.549342	89.35
	WMLRR	0.4174973	0.0279456	0.3756743	64.698374	0.5493742	0.4739754	0.4963865	0.3857491	0.5836492	72.46
	COCA	–	–	–	–	0.6370968	0.2907653	0.3949373	0.3713777	0.6370968	2.26
	SNF.CC	0.6820409	0.4747143	0.2514171	0.2487638	0.7258065	0.4059437	0.4792426	0.4467519	0.7258065	6.46
	SURE	0.3533451	0.0643343	0.8705947	13.6558000	0.8387097	0.5969901	0.5731598	0.6027137	0.8387097	0.34
	CoALa	0.4750780	0.0459589	1.0234149	36.8191400	0.4677419	0.0712289	0.0944394	0.2599910	0.4677419	98.44
	iCluster	0.4133838	0.0248786	0.8147982	40.2522200	0.4435480	0.0270767	0.0364498	0.2436537	0.4435484	175.81
	MiMIC	0.4064141	0.4064141	1.1487670	5.7334720	0.5243129	0.4891097	0.4952194	0.4196284	0.7741935	100.204
	RISynG	0.4824000	0.0701205	0.6639514	14.5428100	**0.8951612**	**0.7191808**	**0.6639029**	**0.6975966**	**0.8951613**	**0.22**
BRCA	SNF	0.4936198	0.3458074	1.2421657	1.7509710	0.6934673	0.4010266	0.4625550	0.3945632	0.6934673	7.88
	CC	0.6653615	0.0702003	0.5397077	13.248510	0.4321608	0.2979244	0.3477434	0.3271579	0.5678392	239.52
	CNMF	0.6428795	0.0423558	0.4194482	33.133060	0.4899497	0.3161555	0.3537982	0.3503934	0.5954774	344.58
	ECMC	–	–	–	–	0.3857462	0.2846732	0.3285674	0.1846376	0.4695832	112.63
	WMLRR	0.4783742	**0.4593621**	**0.1496783**	13.97465	0.5478943	0.2385643	0.3486532	0.2957483	0.4768392	94.32
	COCA	–	–	–	–	0.4045226	0.2211292	0.3145460	0.2821191	0.4824121	12.58
	SNF.CC	**0.8923872**	0.2021579	0.1871910	**0.7428676**	0.4271357	0.4281475	0.4732544	0.4150974	0.7160804	140.11
	SURE	0.2966142	0.0417821	0.9317001	23.00041	0.7562814	0.4798912	0.5063654	0.4569119	0.7562814	2.2
	CoALa	0.3363002	0.0304929	0.8465351	28.189170	0.3919598	0.3907137	0.4685283	0.3923870	0.6758794	737.92
	iCluster	0.3673494	0.0202157	0.9099812	88.604970	0.4798990	0.2900248	0.3659365	0.3185041	0.5000000	145.61
	MiMIC	0.3283235	0.0327848	1.0975070	28.99286	0.3783270	0.4108080	0.4855910	0.3764962	0.6984925	1521.87
	RISynG	0.4296000	0.0459134	0.7135468	33.395980	**0.7613065**	**0.4987076**	**0.5260974**	**0.4716932**	**0.7613065**	**0.56**
OV	SNF	0.4378744	**0.4301527**	1.3849515	**1.7023463**	0.6877637	**0.02818935**	0.0038031	0.5263954	0.6877637	7.06
	CC	0.5903129	0.1032681	**0.4607749**	7.9070535	0.6265823	–0.0323654	**0.0081459**	0.4892693	0.6265823	664.01
	CNMF	0.3538763	0.1154637	0.8634937	435.7459476	0.7046413	0.0278468	0.0031328	0.5436891	0.7046414	263.46
	ECMC	–	–	–	–	0.5694732	0.00047853	0.0001874	0.3857693	0.5849563	172.51
	WMLRR	0.4867543	0.2275846	0.9859643	19.45342	0.5648932	0.0018593	0.0064382	0.4537682	0.6385932	132.12
	COCA	–	–	–	–	0.6370968	− 0.0036014	0.0014148	**0.7846563**	**0.8776371**	4.03
	SNF.CC	0.3957387	0.1673645	0.7284081	2.6021595	0.6793249	0.0141705	0.0010843	0.5211995	0.6793249	62.57
	SURE	0.3222961	0.0111151	0.9517326	326.6007	0.5843258	− 0.0033712	0.0033794	0.440303	0.5063291	1.74
	CoALa	0.3812581	0.0162638	0.9152212	144.9817	0.6540084	0.0078844	0.0004858	0.5005109	0.6540084	1189.76
	iCluster	0.5366303	0.0023473	0.6248983	4335.3610	0.5253164	0.0004292	0.0169881	0.4408962	0.5253165	599.562
	MiMIC	0.3542949	0.0119706	1.1338063	306.19587	**0.8272251**	0.0052532	0.0002227	0.4375362	0.6582278	1678.144
	RISynG	**0.6132400**	0.0043105	0.6058482	877.900712	0.6708865	− 0.0386828	0.0079705	0.5168302	0.6624473	**0.47**
LGG	SNF	0.5552045	0.331413	1.2073461	1.954655	0.6853933	0.3025052	0.3251302	0.4082171	0.6853933	3
	CC	0.6585338	0.2589659	0.3511019	0.7576893	0.8913858	0.6746313	0.6852894	0.6594185	0.8913858	101.73
	CNMF	0.8583117	0.0420026	0.1764646	17.21837	0.5131086	0.1517872	0.2211501	0.3099572	0.5131086	187.31
	ECMC	–	–	–	–	0.6467354	0.5785674	0.7637284	0.5563743	0.6845632	72.51
	WMLRR	0.5648732	0.3365783	0.3486573	72.85743	0.4485673	0.6759743	0.6493754	0.5873549	0.5704737	89.85
	COCA	–	–	–	–	0.6254682	0.2799432	0.3394982	0.4282438	0.6254682	6.31
	SNF.CC	**0.8796842**	**0.5938181**	**0.1291197**	**0.0976532**	0.6853933	0.3025052	0.3251302	0.4082171	0.6853933	152.09
	SURE	0.3834002	0.0950595	0.8558895	6.137831	0.6329588	0.2814732	0.4197476	0.3817919	0.6329588	1.06
	CoALa	0.5106729	0.1025237	0.5474956	6.995131	0.6741573	0.3990866	0.5757631	0.5336079	0.6741573	330.52
	iCluster	0.5840541	0.0145988	0.5850834	158.9932	0.5767790	0.0965103	0.0782154	0.3346215	0.5767790	341.17
	MiMIC	0.5433994	0.0069822	0.7392175	119.9754	0.4472362	0.8460880	0.7071638	0.6392837	0.8812734	487.566
	RISynG	0.4542000	0.1240071	0.7823224	4.904607	**0.9513109**	**0.8752557**	**0.8179747**	**0.8562767**	**0.9513109**	**0.39**
STAD	SNF	0.4390147	**0.4424347**	1.2665290	**1.2660670**	0.3049327	0.0126031	0.0339522	0.2039706	0.3139013	0.94
	CC	0.6517968	0.1587210	0.3735403	2.459431	0.3183857	0.0068337	0.0118838	0.1867525	0.3183857	49.97
	CNMF	**0.9261939**	0.0751574	**0.1297056**	3.592156	0.3049327	0.0055038	0.0085026	0.1800452	0.2959641	124.65
	ECMC	–	–	–	–	0.1847652	0.0018564	0.0035761	0.0174563	0.1785945	84.845
	WMLRR	0.5873752	0.2857689	0.3285963	16.74563	0.1847563	0.0075843	0.0084754	0.0985647	0.1568347	69.213
	COCA	–	–	–	–	0.2600897	0.0055526	0.0211873	0.1730754	0.3004484	3.48
	SNF.CC	0.5330525	0.1745648	0.6411855	2.907296	0.3139013	− 0.0139222	0.0342102	0.1871362	0.3363229	3.33
	SURE	0.3371392	0.0345696	0.7900013	53.22062	0.2825112	0.0250796	0.0243271	0.2243634	0.3991031	0.61
	CoALa	0.3646726	0.0354464	0.7797201	24.92721	0.3318386	0.0080341	0.0319674	0.1969155	0.309417	126.12
	iCluster	0.2700001	0.0551868	1.2110837	26.40223	0.2466360	0.0022785	0.0077143	0.1723892	0.3094170	111.22
	MiMIC	0.3104935	0.0591516	0.9772476	19.93442	0.3854797	0.0096791	0.0101976	0.1493762	0.3766816	214.767
	RISynG	0.3517051	0.0280232	0.7694432	54.50121	**0.3901345**	**0.0260441**	**0.1230677**	**0.2267073**	**0.3901345**	**0.35**

The bold values indicate the best score as reported in the text.

#### Importance of multi-omics data integration

The proposed algorithm RISynG iteratively integrates the relevant subspace of each of the synergy matrices. The relevant subspace corresponds to the *k* largest eigenvectors of the synergy matrices that hold the cluster structure. To exhibit the significance of this iterative integration and the effectiveness of RISynG, it is compared with Spectral clustering performed on individual omics datasets. The results presented in Table 4 show that the proposed algorithm outperforms the individual omic-views in CESC, BRCA, LGG, and STAD datasets for all the external clusters validity indices. In the OV dataset, RISynG outperforms for F-measure, Jaccard, and Purity. However, the miRNA view performs better for ARI and NMI indices. The performance of RISynG is significantly higher than the best individual view in the case of CESC, BRCA, and LGG datasets, irrespective of any indices.

**Figure 3:**
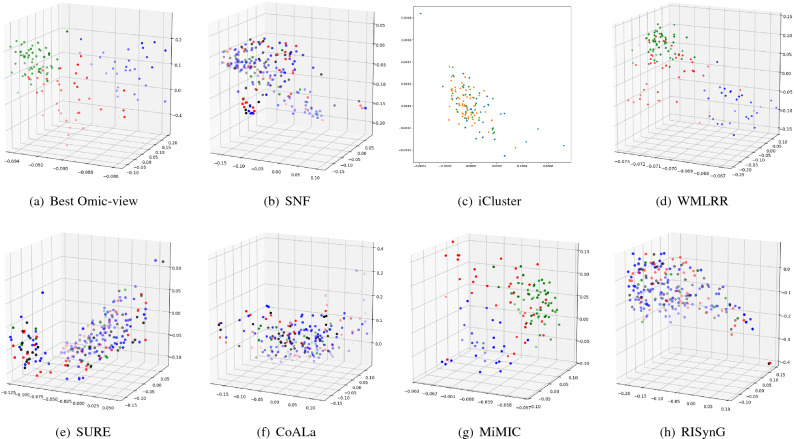
Comparative analysis of different integrative sub-spaces for CESC dataset.

**Figure 4:**
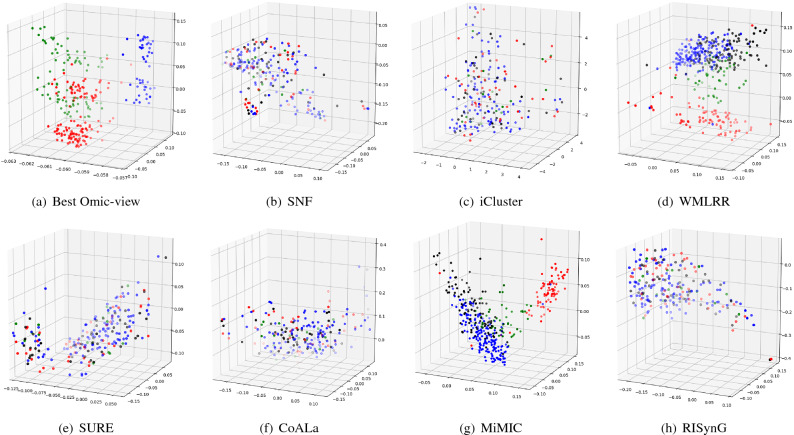
Comparative analysis of different integrative sub-spaces for BRCA dataset.

**Figure 5:**
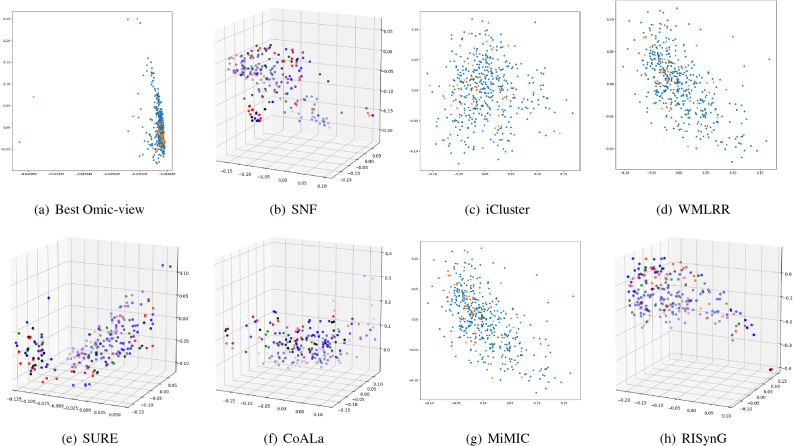
Comparative analysis of different integrative sub-spaces on OV dataset.

**Figure 6:**
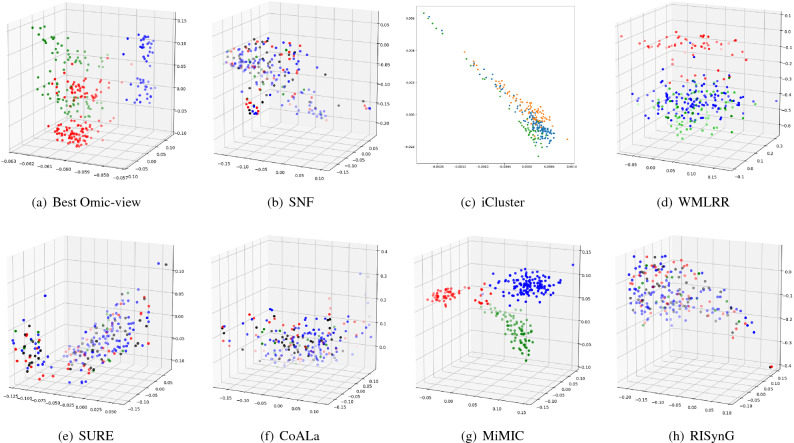
Comparative analysis of different integrative sub-spaces for LGG dataset.

**Figure 7:**
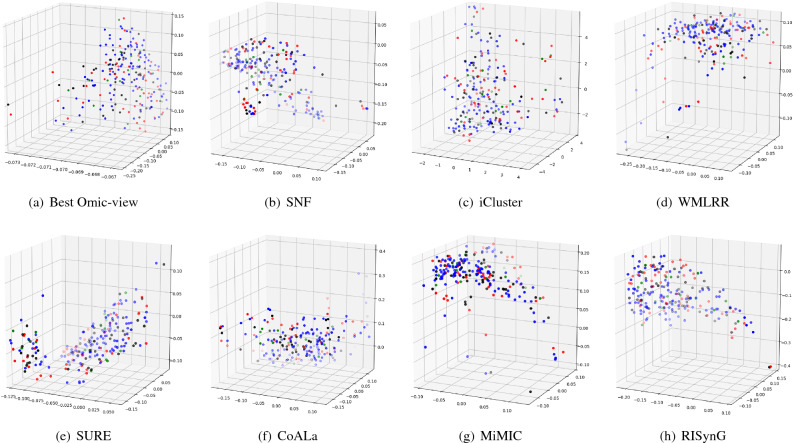
Comparative analysis of different integrative sub-spaces for STAD dataset.

To express the cluster holding capacity of the integrated subspace obtained by the proposed approach, scatter plots for the best *k* dimensions are plotted. The colours in the plots indicate the ground truth (cancer subtypes). Comparative plots are also presented in Figs. 3, 4, 5, 6, and 7 to show that the integrated subspace obtained by RISynG are more informative than other subspace-based integrative-clustering approaches (SNF, SURE, CoALa, iCluster, WMLRR, and MiMIC), for most of the datasets. Comparison with the best individual omic-view (CESC: mRNA, BRCA: metDNA, OV: miRNA, LGG: metDNA, and STAD:miRNA) is also presented to establish the significance of multi-omics data integration performed by the proposed approach. Considering the proposed approach, the scatter plots show that the clusters are well separated in the case of CESC (Fig. 3) and LGG (Fig. 6) datasets. There is a slight overlap between the two groups in BRCA (Fig. 4), but it is better than the other methods. Whereas, for OV (Fig. 5) and STAD (Fig. 7) datasets, the overlap between subtypes is observed in the subspace obtained by all the methods.

**Table 4:** Comparative performance analysis of proposed approach and individual omic-view.

Datasets	Indices	metDNA	miRNA	mRNA	RPPA	RISynG
CESC	F-measure	0.5806452	0.5080645	0.8467742	0.4435484	**0.8951612**
	ARI	0.3642175	0.3632153	0.6854734	0.4398544	**0.7191808**
	NMI	0.5437217	0.5547632	0.6512983	0.6185463	**0.6639029**
	Jaccard	0.6357324	0.5895432	0.6943272	0.6524353	**0.6975966**
	Purity	0.4532324	0.4516432	0.8796542	0.4677554	**0.8951613**
BRCA	F-measure	0.5326633	0.4497487	0.4271357	0.4572864	**0.7613065**
	ARI	0.4297654	0.4197456	0.3458743	0.3982653	**0.4987076**
	NMI	0.5197432	0.5165832	0.5194267	0.5227542	**0.5260974**
	Jaccard	0.4674912	0.3569145	0.3389645	0.3971634	**0.4716932**
	Purity	0.7597435	0.7164987	0.5839622	0.6497312	**0.7613065**
OV	F-measure	*	0.6687764	0.6329114	*	**0.670886**
	ARI	*	**0.0275463**	− 0.0487653	*	− 0.0386828
	NMI	*	**0.0079834**	0.0036542	*	0.0079705
	Jaccard	*	0.4763721	0.3657214	*	**0.5168302**
	Purity	*	0.6547632	0.6585342	*	**0.6624473**
LGG	F-measure	0.7677903	0.4269663	0.5917603	0.4119851	**0.9513109**
	ARI	0.8575432	0.8574643	0.8573215	0.8496432	**0.8752557**
	NMI	0.7589453	0.6965472	0.8143729	0.8054873	**0.8179747**
	Jaccard	0.7565954	0.6986889	0.7548979	0.7765954	**0.8562767**
	Purity	0.6974532	0.9064865	0.8607346	0.8830678	**0.9513109**
STAD	F-measure	*	0.3587444	0.3049327	*	**0.3901345**
	ARI	*	0.0226743	0.0019457	*	**0.0230441**
	NMI	*	0.0224576	0.0219845	*	**0.0230677**
	Jaccard	*	0.1784963	0.2184653	*	**0.2267073**
	Purity	*	0.3794632	0.2845736	*	**0.3901345**

The bold values indicate the best score as reported in the text.

#### Biological analysis

Once the cancer subtypes are obtained, the patient clusters’ molecular characteristic feature is also evaluated to establish their biological relevance. To understand the varying expression of different biomarkers in different subtypes, differential expression analysis (DEA) of miRNAs and mRNAs is performed between the correctly identified groups of patients. A comparative analysis is performed between the true positives and true negatives obtained by all the algorithms. As there are three subtypes in the case of LGG and CESC datasets; therefore, DEA is performed between three pairs (considering all possible pairs). Similarly, in the case of STAD and BRCA datasets, since there are four subtypes, DEA is performed for six pairs, and for the OV dataset, there are two subtypes; therefore, DEA is performed for one pair. R package Limma^64^ is used to perform DEA. miRNAs and mRNAs having Bejamini-Hochberg false discovery rate adjusted *p*-value $< 0.05$ are considered as differentially expressed. Number of differentially expressed biomarkers obtained from different groups in CESC, BRCA, OV, LGG, and STAD datasets are reported in Tables 5, 6, 7, 8, and 9 respectively. To further explore and highlight the biological knowledge and process-specific functioning of the identified sets of differentially expressed biomarkers, different types of enrichment analyses are also performed, considering the hundred most differentially expressed biomarkers in each case.

#### Biological enrichment analyses

The first analysis is Pathway enrichment analysis (PEA). It explores the mechanistic insight into the set of differentially expressed biomarkers. It helps identify those biological pathways enriched in a set of biomarkers more than expected by chance. The second one is Biological process enrichment analysis (BPEA). It helps characterize the relationship between genes or miRNAs by specifically annotating them to associated biological processes. It helps identify the over-represented biological processes in our list, which can help evaluate the biological significance of the obtained cancer subtypes. Furthermore, the third one is Disease ontology enrichment analysis (DOEA). Disease Ontology (DO) helps map the relevance of cancer subtypes identified from high-throughput data to clinical relevance. In this study, the R package, clusterProfiler^65^ and DIANA Tools mirPath v.3^66^ are used for performing PEA and BPEA for genes and miRNAs, respectively, and R package DOSE^67^ is used to perform DOEA for the genes. The top 100 differentially expressed biomarkers are passed to these tools. In some cases, if the number of differentially expressed biomarkers is less than 100, then all of them are used. KEGG database is selected for PEA^68^. All the pathway terms associated with the set of biomarkers having false discovery rate adjusted *p*-value $< 0.05$ (significant pathway terms) are only considered. Suppose any differentially expressed biomarker sets are not associated with significant KEGG pathway terms. In that case, that set is said to be not biologically relevant with respect to KEGG pathway terms. Similarly, all the biological process (BP) terms associated with the set of biomarkers having a false discovery rate adjusted *p*-value $< 0.05$ (significant pathway terms) are only considered. If any of the differentially expressed biomarker sets are not associated with significant BP terms, that set is said to be not biologically relevant with respect to BP terms. In DOEA, semantic similarities between DO terms and genes are calculated that help explore the similarities of diseases and gene functions from a disease perspective. The output of DOES has associated disease terms. A gene set is said to be enriched with DO terms if the terms obtained by its DOEA have a false discovery rate corrected *p*-value $<0.05$.

For the quantification of KPEA, BPEA, and DOEA, respective enrichment scores^69^, and annotation ratios^69^ are calculated. The higher the value of these scores better is the enrichment; hence, the more biologically significant the differentially expressed biomarkers are, the better the cancer sub-typing. Following are the equations for these scores:

47 $$\begin{aligned} BPES= & {} \frac{1}{T} \sum _{t=1}^{T}-log_{10}(p-value_{t}), \end{aligned}$$

48 $$\begin{aligned} AR= & {} \frac{1}{T \times G} \sum _{i=1}^{T}g_{i}. \end{aligned}$$

Here, T denotes the number of significant pathway/BP/terms associated with a set of differentially expressed genes or miRNAs between two cancer subtypes identified by any clustering approaches. G denotes the total number of genes given to clusterProfiler for the enrichment analysis, and g denotes the gene count associated with a pathway/BP/DO term. Comparative analysis of the cancer subtypes obtained by the proposed approach and other existing algorithms are performed and the associated quantitative indices are reported in Tables 5, 6, 7, 8, and 9. Some of the differentially expressed miRNAs or mRNAs have no associated significant terms; therefore, there is no scope for calculating the quantitative indices. Also, in some cases, there are no differentially expressed biomarkers. All these cases are represented by $*$ in the tables.

**Figure 8:**
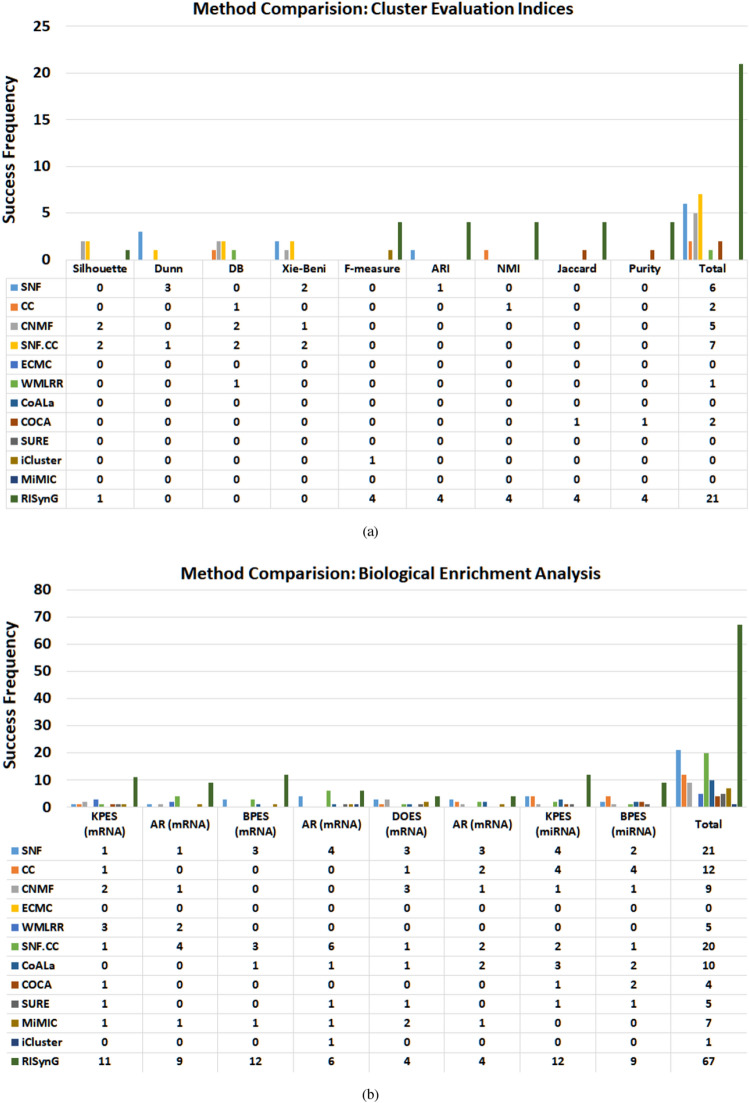
Method comparison.

To compare the effectiveness of the proposed approach with the other algorithms in this study, the overall performance of all the methods is also evaluated. When all the five cancer datasets are considered together, the proposed approach outperforms concerning both cluster evaluation indices and biological enrichment analysis, as shown in Fig. 8. The analysis is performed by considering the success frequency (number of times a method scored the highest value for respective indices when all the cases in all the cancer types are considered). The success frequency shows that the proposed approach outperforms when cluster validity indices are considered by scoring maximum values for 21 times, followed by SNF.CC (7), SNF (6), CNMF (5), CC (2), COCA (2), and WMLRR (1). Similarly, suppose the methods are ranked considering the success frequency for quantitative indices calculated for biological enrichment analysis. In that case, the proposed approach will again stand first by scoring the maximum value 67 times, followed by SNF (21), SNF.CC (20), CC (12), CoALa (10), CNMF (9), MiMIC (7), SURE (5), WMLFF (5), COCA (4), and iCluster (1). If the cluster validity indices are looked upon individually, the proposed approach also outperforms with respect to F-measure, ARI, NMI, Jaccard index, and Purity. Considering the indices for biological enrichment individually, the proposed algorithm again outperforms with respect to all the indices except for AR for BPES for mRNA enrichment, where it stands second.

**Table 5:** Comparative biological analysis of CESC dataset.

CESC classes	Methods	mRNA enrichment analysis						miRNA enrichment analysis		Number of differentially expressed	
		KPES	AR	BPES	AR	DOES	AR	KPES	BPES	mRNAs	miRNAs
Keratin low squamous vs keratin high squamous	SNF	2.253130	**0.013174**	2.124593	**0.014242**	1.492051	0.002597	*	*	811	9
	CC	3.688672	0.001579	2.227388	0.002396	1.533960	0.008514	*	*	829	5
	CNMF	1.533344	0.006379	2.021209	0.001684	1.383268	0.007361	*	*	719	3
	ECMC	1.658394	0.003756	0.002134	0.006945	0.385745	0.003684	*	*	529	6
	WMLRR	*	*	*	*	*	*	*	*	34	4
	SNF.CC	2.253130	**0.013174**	2.124593	**0.014242**	1.492051	0.002597	*	*	811	9
	CoALa	1.650947	0.004808	2.381972	0.006842	1.397646	**0.010533**	*	*	632	5
	COCA	2.742077	0.010161	1.540280	0.010722	1.764804	0.000658	*	*	529	1
	SURE	10.322586	0.005417	4.595299	0.009796	1.656721	0.007143	*	*	915	7
	iCluster	6.456398	0.002674	3.564834	** 0.014240**	1.465782	0.003657	3.459342	1.45698	939	92
	MiMIC	5.739475	0.003295	2.593754	0.004743	1.285647	0.004738	*	*	643	6
	RISynG	**19.023039**	0.003559	**4.483853**	0.007835	**1.993933**	0.003492	**4.730997**	**3.702912**	924	13
Keratin low squamous vs adenocarcinoma	SNF	2.228672	0.010167	2.060098	0.007604	1.586351	**0.030129**	**2.839092**	3.702912	748	23
	CC	1.524904	0.004545	2.005994	0.003854	1.396591	0.011918	**2.839092**	3.510903	545	19
	CNMF	1.837035	0.001731	*	*	**1.985854**	0.001143	2.838999	3.510903	398	13
	ECMC	0.568364	0.003956	2.967456	0.002756	1.486344	0.018934	1.459674	1.498264	544	54
	WMLRR	1.486745	0.002758	1.584754	0.001845	*	*	*	*	66	12
	SNF.CC	2.228672	0.003585	2.060098	0.007604	1.586351	**0.030129**	2.838999	3.510903	748	23
	CoALa	*	*	1.819866	0.004375	1.530370	0.029351	2.838999	3.702912	489	17
	COCA	2.095093	0.005333	2.266488	0.006105	1.812902	0.011081	2.838999	3.702912	600	14
	SURE	1.986232	0.002903	1.825145	**0.007835**	1.773099	0.021688	2.838999	4.853914	826	24
	iCluster	1.657453	0.003746	2.567439	0.006345	*	*	2.838998	3.675432	842	14
	MiMIC	1.698345	0.003521	2.674931	0.005392	1.436284	0.016385	*	*	819	2
	RISynG	**2.902596**	**0.010166**	**4.699835**	0.000833	*	*	2.186029	**7.052373**	966	34
Keratin high squamous vs adenocarcinoma	SNF	*	*	**3.405602**	0.004409	*	*	**2.838998**	7.034831	774	38
	CC	*	*	2.306625	0.004421	1.580965	**0.006619**	**2.838999**	7.034831	652	27
	CNMF	*	*	2.271344	0.003191	**1.822238**	0.004493	**2.838998**	7.034831	478	23
	ECMC	0.568364	0.003956	2.967456	0.002756	1.486344	0.018934	1.459674	1.498264	544	54
	WMLRR	1.385674	0.859432	1.674834	0.002856	1.495733	0.001745	*	*	633	17
	SNF.CC	*	*	1.701994	0.004409	*	*	**2.838998**	7.034831	774	39
	CoALa	*	*	2.934221	0.005957	1.505341	0.006575	*	*	535	18
	COCA	*	*	3.229027	0.004839	*	*	**2.838998**	7.105402	553	20
	SURE	*	*	2.931849	0.003871	1.432160	0.001884	**2.838998**	**7.819319**	790	52
	iCluster	*	*	2.627392	0.005734	*	*	1.645321	3.458233	637	96
	MiMIC	**2.564893**	** 1.453983**	2.845623	0.001634	*	*	*	*	412	0
	RISynG	*	*	**3.405602**	**0.009148**	*	*	**2.838998**	**7.819319**	951	63

The bold values indicate the best score as reported in the text.

**Table 6:** Comparative biological analysis of BRCA dataset.

BRCA classes	Methods	mRNA enrichment analysis						miRNA enrichment analysis		Number of differentially expressed	
		KPES	AR	BPES	AR	DOES	AR	KPES	BPES	mRNAs	miRNAs
Luminal A vs luminal B	SNF	1.461915	0.002553	1.385690	0.000306	**2.477131**	0.001970	2.372875	5.449253	1198	106
	CC	1.478030	0.000755	1.365630	0.000306	*	*	2.632318	4.086584	864	49
	CNMF	**1.959958**	**0.0024**	*	*	2.461538	0.003279	*	**6.847070**	367	3
	ECMC	*	*	*	*	*	*	*	*	21	7
	WMLRR	*	*	*	*	*	*	*	*	0	5
	SNF.CC	1.613135	0.001379	2.372456	0.006186	2.135156	0.082714	3.676487	6.071079	236	45
	CoALa	1.418795	0.001311	**2.637358**	**0.006562**	2.162850	**0.089027**	**6.361579**	5.144748	225	36
	COCA	*	*	*	*	*	*	*	*	0	0
	SURE	*	*	*	*	*	*	*	*	367	0
	iCluster	*	*	*	*	*	*	*	*	1186	89
	MiMIC	*	*	1.598348	0.0002643	*	*	*	*	586	14
	RISynG	*	*	*	*	*	*	*	*	0	0
Luminal A vs Her-2 enriched	SNF	*	*	*	*	*	*	2.143165	5.339598	1522	181
	CC	*	*	*	*	1.679493	0.001791	1.975801	5.557729	1429	129
	CNMF	*	*	*	*	*	*	2.316795	*	1501	181
	SNF.CC	1.528961	0.003288	2.484664	0.007766	**2.472259**	**0.081621**	4.772078	5.635141	204	34
	ECMC	1.645964	0.002194	0.763859	0.002856	*	*	*	*	516	19
	WMLRR	1.456983	0.0018465	1.678453	0.003956	1.649663	0.045843	3.856433	4.856377	1125	11
	CoALa	2.053401	0.003889	1.352696	0.004352	2.418967	0.066857	**5.981546**	5.361591	175	39
	COCA	*	*	*	*	*	*	2.182702	**6.704499**	1419	175
	SURE	*	*	*	*	*	*	2.143102	5.339598	1501	179
	iCluster	*	*	*	*	*	*	3.185943	4.675320	1486	177
	MiMIC	1.957394	0.002845	1.749245	0.002945	1.849382	0.025383	2.795843	3.738291	1891	181
	RISynG	**2.069400**	**0.004285**	**2.552696**	**0.008152**	*	*	2.143165	5.339598	1555	181
Luminal A vs basal like	SNF	*	*	1.968809	0.001237	1.671238	0.014714	*	6.208960	1514	134
	CC	*	*	*	*	2.650350	0.001944	2.099859	**7.595675**	1426	115
	CNMF	1.527132	0.001176	*	*	2.315198	0.002143	2.027684	4.551682	1420	111
	ECMC	*	*	*	*	*	*	*	*	112	0
	WMLRR	*	*	1.894784	0.001768	*	*	*	*	253	16
	SNF.CC	2.301212	0.001296	**2.320499**	**0.011444**	1.586312	0.004521	*	*	155	5
	CoALa	*	*	*	*	*	*	*	*	0	0
	COCA	*	*	1.867577	0.001563	2.715869	0.001972	2.182702	6.704499	1255	115
	SURE	*	*	*	*	*	*	2.463865	4.086584	1420	66
	iCluster	1.567384	0.006354	*	*	*	*	1.945638	5.639721	1530	133
	MiMIC	1.563985	0.005932	1.486932	0.003285	1.748234	0.032863	0.638291	0.638294	512	110
	RISynG	**2.318606**	**0.02**	*	*	**3.529331**	**0.035571**	**2.518622**	4.086584	1153	61
Luminal B vs Her-2 enriched	SNF	1.556018	0.006964	1.895925	0.001087	**1.711230**	0.004521	2.702356	**7.005326**	1074	111
	CC	1.848055	0.001961	*	*	1.611678	**0.043432**	**2.893976**	2.140340	734	27
	CNMF	*	*	*	*	*	*	*	*	32	0
	SNF.CC	1.768648	0.001296	**2.439050**	**0.004365**	1.505375	0.037612	2.702356	**7.005326**	1173	119
	ECMC	1.245874	0.001749	0.846396	0.002469	1.285749	0.0175635	2.489275	4.867463	972	94
	WMLRR	**2.485737**	**0.185483**	1.946385	0.000174	0.946285	0.02857	1.674927	4.683956	1432	116
	CoALa	1.516468	0.000806	2.296736	0.002857	1.422305	0.012857	2.702356	**7.005326**	1209	113
	COCA	*	*	*	*	*	*	*	*	0	0
	SURE	*	*	*	*	*	*	*	*	32	0
	iCluster	*	*	*	*	*	*	2.567493	6.978346	135	90
	MiMIC	*	*	*	*	*	*	*	*	65	4
	RISynG	*	*	*	*	*	*	*	*	0	0
Luminal B vs basal like	SNF	**2.128049**	**0.0156**	*	*	**1.819763**	**0.060895**	**1.838692**	**3.481747**	1096	99
	CC	1.658311	0.006875	1.370137	0.000313	1.705471	0.044328	*	2.140340	897	66
	CNMF	*	*	*	*	*	*	*	*	15	0
	SNF.CC	2.118175	0.008654	**2.476302**	**0.013333**	1.695800	0.009855	*	*	158	0
	ECMC	*	*	*	*	*	*	*	*	54	0
	WMLRR	1.756342	0.001856	1.674834	0.002849	*	*	*	*	856	0
	CoALa	*	*	*	*	*	*	*	*	0	0
	COCA	*	*	*	*	*	*	*	*	0	0
	SURE	*	*	*	*	*	*	*	*	15	0
	iCluster	*	*	*	*	*	*	*	*	1051	57
	MiMIC	*	*	*	*	*	*	*	*	84	0
	RISynG	*	*	*	*	*	*	*	*	0	0
Her-2 Enriched vs basal like	SNF	1.655414	0.003158	3.157786	0.002188	2.523476	0.110366	*	*	1120	152
	CC	1.920310	0.000714	2.702511	0.001505	1.800734	0.008451	*	*	450	11
	CNMF	2.143113	0.002097	3.196699	0.015612	2.245955	0.056154	2.749089	46.059107	1057	147
	ECMC	1.658453	0.006856	1.475986	0.001845	1.678432	0.027923	1.48234	4.674983	943	89
	WMLRR	1.287463	0.004738	2.476993	0.004845	1.283622	0.001745	1.945673	4.687384	1921	112
	SNF.CC	2.558315	0.001212	3.033794	0.018085	1.774396	0.021528	*	*	112	0
	CoALa	*	*	*	*	*	*	*	*	0	0
	COCA	1.830650	0.002623	1.691438	0.003438	*	*	2.382039	4.804657	362	231
	SURE	*	*	*	*	*	*	2.615351	4.166632	1057	49
	iCluster	2.598346	0.002864	3.167489	0.017456	*	*	2.498563	3.758943	1175	128
	MiMIC	2.649362	0.003785	2.749734	0.001373	*	*	*	*	278	0
	RISynG	**2.844053**	**0.013333**	**3.384443**	**0.024848**	**2.307443**	**0.125301**	**2.938526**	**6.524382**	900	60

The bold values indicate the best score as reported in the text.

**Table 7:** Comparative biological analysis of OV dataset.

OV classes	Methods	mRNA enrichment analysis						miRNA enrichment analysis		Number of differentially expressed	
		KPES	AR	BPES	AR	DOES	AR	KPES	BPES	mRNA	miRNA
Vs Neoplasm histological grade 3 Neoplasm histological grade 2	SNF	1.898710	0.002381	**2.061189**	**0.001053**	*	*	*	7.871558	268	230
	CC	*	*	*	*	*	*	*	*	0	0
	CNMF	*	*	*	*	*	*	*	*	0	0
	ECMC	*	*	*	*	*	*	*	*	54	0
	WMLRR	*	*	2.148563	0.000584	*	*	*	*	123	11
	CoALa	4.675430	0.003810	*	*	*	*	2.156299	10.129178	214	178
	SNF.CC	5.155090	0.003125	1.354431	0.000947	*	*	*	*	331	203
	COCA	*	*	*	*	*	*	*	*	0	0
	SURE	1.640585	0.010000	*	*	*	*	2.456219	5.679494	485	19
	iCluster	5.864734	0.016740	1.256483	0.001030	*	*	1.178456	7.457563	664	228
	MiMIC	5.327456	0.014639	1.645294	0.000143	**2.745934**	**0.763549**	2.458396	7.935281	981	172
	RISynG	**7.004104**	**0.020000**	*	*	*	*	**2.715358**	**13.354195**	256	209

The bold values indicate the best score as reported in the text.

**Table 8:** Comparative biological analysis of LGG dataset.

LGG classes	Methods	mRNA enrichment analysis						miRNA enrichment analysis		Number of differentially expressed	
		KPES	AR	BPES	AR	DOES	AR	KPES	BPES	mRNAs	miRNAs
IDH mutation without 1p/19q codeletion vs IDH mutation with 1p/19q codeletion	SNF	2.216865	0.004390	1.712976	0.010313	1.518906	**0.007586**	2.623947	7.443965	1530	180
	CC	2.060736	0.002167	1.828709	0.002371	*	*	2.381158	5.218678	1394	185
	CNMF	2.713893	0.004400	2.184500	0.023895	**1.737469**	0.003881	2.377525	6.805268	1475	197
	ECMC	2.569342	0.017453	2.983657	0.011547	1.385943	0.004856	3.684935	6.956385	1632	87
	WMLRR	2.957453	0.003758	2.567396	0.003854	0.946583	0.004867	2.956486	3.574869	943	114
	SNF.CC	2.158081	0.001250	1.857196	**0.012580**	*	*	2.433907	6.981976	1346	186
	CoALa	*	*	*	*	*	*	*	*	0	0
	COCA	1.868207	0.005893	1.445307	0.002021	*	*	3.009416	**8.168954**	1091	209
	SURE	1.667323	0.000862	2.918052	0.002421	*	*	1.722047	2.988518	1022	46
	iCluster	2.6547342	0.00345628	2.674538	0.0045638	1.265483	0.0034562	3.256437	3.756417	1445	180
	MiMIC	2.983452	0.0352854	1.845372	0.011845	1.436956	0.004637	3.756281	5.342743	1223	124
	RISynG	**3.015521**	**0.047500**	**3.165604**	0.000729	*	*	**5.831749**	2.118806	1286	129
IDH mutation without 1p/19q codeletion vs wild type IDH subtype	SNF	5.334880	0.002250	2.177240	0.001848	*	*	2.191319	4.547026	1380	151
	CC	10.753373	0.002857	4.952588	0.002444	*	*	**3.276499**	3.693358	1334	255
	CNMF	3.828751	0.001905	1.827162	0.001739	*	*	2.640297	6.127825	1410	152
	ECMC	*	*	*	*	*	*	*	*	85	16
	WMLRR	17.47594	0.028475	3.674834	0.017385	*	*	*	*	323	35
	SNF.CC	2.313690	**0.030833**	2.004395	**0.030232**	*	*	*	*	1151	0
	CoALa	2.308687	0.005500	1.676198	0.012824	**1.835847**	**0.002712**	2.134683	6.374528	995	120
	COCA	*	*	*	*	*	*	2.191284	4.547026	1333	147
	SURE	8.396959	0.003750	4.759305	0.003146	*	*	2.598835	5.097597	1207	90
	iCluster	7.753974	0.002634	3.378298	0.004573	*	*	1.456382	4.634529	1476	203
	MiMIC	5.437984	0.016453	4.863478	0.024537	0.562849	0.000174	*	*	312	0
	RISynG	**16.665788**	0.002553	**6.554131**	0.001868	*	*	2.957544	**7.361838**	1299	162
IDH mutation with 1p/19q codeletion vs wild type IDH subtype	SNF	4.872952	0.001818	2.830515	0.003646	*	*	2.433906	6.981966	1433	186
	CC	1.770337	0.004118	1.845716	0.003118	2.150893	0.008475	**2.532364**	4.105869	1463	154
	CNMF	4.479526	0.004310	2.153374	0.008229	*	*	1.774068	5.301943	1299	177
	ECMC	*	*	2.584763	0.028496	*	*	*	*	122	5
	WMLRR	**5.968354**	0.005846	1.956384	0.017485	1.956486	1.956738	1.745867	5.956385	1765	165
	SNF.CC	2.669711	**0.009000**	2.035048	0.026484	1.985843	0.009483	2.623947	**7.443965**	888	180
	CoALa	*	*	*	*	*	*	*	*	0	0
	COCA	2.144910	0.007805	1.803831	0.020526	1.611270	0.006102	2.301836	7.175007	1320	195
	SURE	1.456283	0.002459	2.830515	0.004409	2.543007	0.009483	**2.532364**	4.105869	1321	118
	iCluster	1.456735	0.001645	1.456872	0.001674	*	*	1.456380	4.764537	1172	148
	MiMIC	3.956382	0.004281	2.459216	**0.038294**	2.453856	0.000352	1.463823	3.785645	1145	193
	RISynG	1.761283	0.002683	**2.835968**	0.004409	**2.866000**	**0.015172**	2.355656	5.218530	1321	144

The bold values indicate the best score as reported in the text.

**Table 9:** Comparative biological analysis of STAD dataset.

STAD classes	Methods	mRNA enrichment analysis						miRNA enrichment analysis		Number of differentially expressed	
		KPES	AR	BPES	AR	DOES	AR	KPES	BPES	mRNAs	miRNAs
MSI vs EBV	SNF	*	*	**2.947976**	**0.007778**	*	*	3.886525	5.339598	51	139
	CC	*	*	*	*	*	*	3.475801	5.557729	0	117
	CNMF	*	*	*	*	*	*	2.286346	5.274368	0	173
	ECMC	*	*	1.486745	0.001754	*	*	*	*	76	19
	WMLRR	*	*	*	*	*	*	*	*	0	0
	SNF.CC	**1.641207**	**0.009500**	1.808831	0.003590	*	*	4.663422	5.635141	51	308
	CoALa	*	*	*	*	*	*	2.167354	5.631591	0	130
	COCA	*	*	*	*	*	*	4.182702	5.649816	0	130
	SURE	*	*	*	*	*	*	2.143102	5.339598	1939	502
	iCluster	*	*	*	*	*	*	*	*	0	3
	MiMIC	*	*	*	*	*	*	*	*	0	0
	RISynG	*	*	*	*	*	*	**5.981546**	**6.704499**	0	138
MSI vs CIN	SNF	3.206072	0.006809	1.919825	0.012532	2.101852	0.005965	2.640297	4.547026	88	213
	CC	2.463472	0.004118	1.677523	0.014000	1.732361	0.004000	1.191319	3.593216	233	147
	CNMF	*	*	1.412950	0.005217	*	*	2.275633	3.547026	128	74
	ECMC	3.867453	0.002756	1.584732	0.001748	1.093748	0.004856	0.083956	3.956483	954	99
	WMLRR	1.856093	0.003756	2.256943	0.018463	2.056783	0.005734	1.756398	5.935744	432	118
	SNF.CC	1.517881	0.001111	1.376410	0.001970	*	*	1.337563	5.097597	88	388
	CoALa	3.152809	0.006471	2.094399	0.013291	2.033564	0.004918	2.718835	5.127825	288	219
	COCA	3.152809	0.006471	2.094399	0.013291	2.033564	0.004918	0.387436	4.447824	288	219
	SURE	3.494658	0.005882	2.250916	0.013377	**2.194897**	0.002586	2.191284	5.376595	333	239
	iCluster	3.679345	0.003785	1.764538	0.003856	1.456396	0.006742	1.645297	4.762394	130	260
	MiMIC	4.678234	0.006453	1.845632	**0.0267453**	2.074592	0.005643	1.645372	4.756382	204	88
	RISynG	**5.672025**	**0.007447**	**2.361069**	0.011270	1.859974	**0.008033**	**2.957544**	**6.374528**	168	216
MSI vs GS	SNF	1.625946	0.003333		0.013099	1.667743	0.013158	**3.534893**	2.140340	46	128
	CC	4.408086	0.006061	1.929352	0.014853	**2.178884**	0.008039	2.674398	2.238740	297	108
	CNMF	2.923027	0.001667	2.062190	0.016000	1.778082	**0.015600**	2.226550	2.347540	90	17
	ECMC	4.756823	0.002745	0.587384	0.027485	1.748396	0.003856	2.567498	2.986482	543	164
	WMLRR	3.056845	0.003856	0.574983	0.003758	2.489564	0.002745	1.859644	3.975844	643	219
	SNF.CC	2.142436	0.007500	1.771466	0.000811	*	*	*	*	46	284
	CoALa	3.008414	0.007143	1.836751	0.015467	1.701261	0.014107	2.615463	**4.376543**	220	129
	COCA	3.008414	0.007143	1.836751	0.015467	1.701261	0.014107	2.615463	**4.376543**	220	129
	SURE	3.245879	0.004889	1.889092	0.013288	2.090617	0.010727	3.286473	2.238740	124	42
	iCluster	3.651936	0.014563	0.674328	0.003549	1.845362	0.007453	3.254698	3.934657	27	112
	MiMIC	2.145936	0.004563	1.434328	**0.141283**	1.732362	0.005453	*	*	116	0
	RISynG	**5.266844**	**0.018500**	**1.960004**	0.001935	*	*	3.223198	3.674321	39	303
EBV vs CIN	SNF	*	*	*	*	*	*	1.481368	3.654953	0	237
	CC	*	*	*	*	*	*	2.228647	**4.578635**	0	214
	CNMF	*	*	*	*	*	*	2.193468	1.645390	0	35
	ECMC	*	*	*	*	*	*	*	*	0	15
	WMLRR	**2.856748**	**0.947567**	0.364867	0.001985	*	*	*	*	156	65
	SNF.CC	*	*	*	*	*	*	*	*	0	0
	CoALa	*	*	*	*	*	*	1.774584	2.984563	33	236
	COCA	*	*	*	*	*	*	2.193468	2.537548	33	236
	SURE	*	*	1.375449	0.002295	*	*	2.306584	2.756483	1939	502
	iCluster	*	*	*	*	*	*	*	*	15	0
	MiMIC	*	*	*	*	*	*	*	*	7	0
	RISynG	*	*	**1.643941**	**0.002857**	*	*	**2.312785**	2.865474	18	139
EBV vs GS	SNF	*	*	*	*	*	*	1.481368	3.654953	0	119
	CC	*	*	*	*	*	*	2.228647	**4.578635**	0	102
	CNMF	*	*	*	*	*	*	2.193468	1.645390	0	31
	ECMC	*	*	*	*	*	*	*	*	17	6
	WMLRR	*	*	0.678465	1.298456	*	*	*	*	119	12
	SNF.CC	*	*	*	*	*	*	*	*	0	42
	CoALa	*	*	*	*	*	*	1.774584	2.984563	0	134
	COCA	*	*	*	*	*	*	2.193468	2.537548	0	134
	SURE	*	*	1.375449	0.002295	*	*	2.306584	2.756483	1939	502
	iCluster	*	*	*	*	*	*	*	*	70	0
	MiMIC	1.274567	0.006543	1.274973	0.009451	1.374516	0.004373	2.264983	3.564832	110	67
	RISynG	**1.424199**	**0.010000**	**1.686765**	**0.008333**	*	*	**2.312785**	2.865474	6	60
CIN vs GS	SNF	*	*	*	*	*	*	2.546392	3.058563	0	255
	CC	1.609349	**0.007222**	*	*	*	*	2.964875	3.337658	247	39
	CNMF	**2.689639**	0.004063	1.780668	0.007778	*	*	*	*	229	17
	ECMC	0.856377	0.003756	1.658499	0.004867	*	*	2.568493	2.583993	132	213
	WMLRR	*	*	*	*	*	*	*	*	0	32
	SNF.CC	*	*	*	*	*	*	1.948756	2.354654	0	212
	CoALa	*	*	1.637374	0.000625	*	*	0.864563	2.904373	64	222
	COCA	*	*	1.637374	0.000625	*	*	1.084653	3.569463	64	222
	SURE	1.967703	0.004737	1.455467	0.002667	*	*	3.564875	4.724974	98	325
	iCluster	2.645382	0.0037845	1.956734	0.000376	*	*	1.567354	2.645983	69	14
	MiMIC	2.534967	0.005378	1.432134	0.001453	*	*	2.134975	2.195342	112	54
	RISynG	*	*	**2.947976**	**0.010656**	*	*	**3.956474**	**4.765984**	92	283

The bold values indicate the best score as reported in the text.

#### Overlap analysis

The hundred most differentially expressed genes between all the subtypes-pairs in cervical cancer that RISynG and the other methods identified are explored further for experimental support. The genes are analyzed based on the degree of overlap with known cervical cancer genes that are experimentally validated. The Cervical Cancer Gene Database (CCDB)^70^ is used for finding the overlap. It is a manually curated catalog of experimentally validated genes involved in the different stages of cervical carcinogenesis. All the up-regulated and down-regulated genes in cervical cancer with evidence from the published literature available in CCDB are considered for this analysis. 367 genes are reported in CCDB that are differentially expressed in cervical cancer. This list contains 185 genes from a total number of 2000 genes that are used for cancer subtype identification in this study. The statistical significance of the overlap analysis is reported in Table 10. In total, 30 genes out of 222 identified from the proposed approach overlap with cervical cancer-related genes. This is the maximum overlap when compared with the other methods. Fisher’s exact test is used here to find the statistical significance of the contingency table created from the overlap analysis in Table 10 for different algorithms. At 95% confidence, it is observed that only the genes identified by the proposed approach have significant overlap with experimentally validated genes curated from literature with a *p*-value of 0.026. Therefore, it indicates that the proposed approach has the potential to identify clinically important subtypes of cancer that have a characteristic molecular signature.

**Table 10:** Overlap with experimentally validated gene-list.

Methods		Yes	no	Total	*p*-value
SNF	Yes	22	202	224	0.715
	No	163	1613	1776	
CC	Yes	20	198	218	1.000
	No	165	1617	1782	
CNMF	Yes	26	197	223	0.219
	No	159	1618	1777	
ECMC	Yes	9	272	281	0.053
	No	176	1543	1719	
WMLRR	Yes	13	156	169	0.578
	No	172	1659	1831	
SNF.CC	Yes	22	202	224	0.715
	No	163	1613	1776	
CoALa	Yes	19	200	219	0.902
	No	166	1615	1781	
COCA	Yes	23	194	217	0.457
	No	162	1621	1783	
SURE	Yes	18	207	225	0.543
	No	167	1608	1775	
iCluster	Yes	17	205	222	0.460
	No	168	1610	1778	
MiMIC	Yes	19	216	235	0.631
	No	166	1599	1765	
RISynG	Yes	30	192	222	0.026
	No	155	1623	1778	
Total		185	1815	2000	

## Conclusion

The present study describes a method named RISynG that efficiently identifies cancer subtypes. Cancer subtypes identification can facilitate cancer diagnosis and therapy. It is one of the vital components of the precision medicine framework. The main contributions of this study are: (1) Development of an integrative clustering method for multi-view omics data. (2) Demonstration of the effectiveness of the proposed method over other methods. (3) Establishing biological relevance for the obtained results.

## Data Availability

The python scripts for RISynG and the pre-processed sample-matched datasets are available at http://home.iitj.ac.in/~sushmitapaul/CBL/code/RISynG.zip.

## References

[1] Stingl J, Caldas C (2007). Molecular heterogeneity of breast carcinomas and the cancer stem cell hypothesis. Nat. Rev. Cancer.

[2] Liang M, Li Z, Chen T, Zeng J (2015). Integrative data analysis of multi-platform cancer data with a multimodal deep learning approach. IEEE/ACM Trans. Comput. Biol. Bioinform..

[3] Tomczak K, Czerwińska P, Wiznerowicz M (2015). The Cancer Genome Atlas (TCGA): An immeasurable source of knowledge. IEEE/ACM Trans. Comput. Biol. Bioinform..

[4] Therese S (2001). Gene expression patterns of breast carcinomas distinguish tumor sub classes with clinical implications. Proc. Natl. Acad. Sci. U.S.A..

[5] Bhattacharjee A (2001). Classification of human lung carcinomas by mRNA expression profiling reveals distinct adenocarcinoma sub classes. Proc. Natl. Acad. Sci. U.S.A..

[6] Monti S, Tamayo P, Mesirov J, Golub T (2003). Consensus clustering: A resampling-based method for class discovery and visualization of gene expression microarray data. Mach. Learn..

[7] Teschendorff AE, Miremadi A, Pinder SE, Ellis IO, Caldas C (2007). An immune response gene expression module identifies a good prognosis subtype in estrogen receptor negative breast cancer. Genome Biol..

[8] Zhang W, Feng H, Wu H, Zheng X (2017). Accounting for tumor purity improves cancer subtype classification from DNA methylation data. Bioinformatics.

[9] Network CGA (2012). Comprehensive molecular portraits of human breast tumours. Nature.

[10] Network CGA (2012). Comprehensive molecular characterization of human colon and rectal cancer. Nature.

[11] Hoadley KA (2014). Multiplatform analysis of 12 cancer types reveals molecular classification within and across tissues of origin. Cell.

[12] Gabasova E, Reid J, Wernisch L (2017). Clusternomics: Integrative context-dependent clustering for heterogeneous datasets. PLos Comput. Biol..

[13] Bo W (2014). Similarity network fusion for aggregating data types on a genomic scale. Nat. Methods.

[14] Shen R, Olshen AB, Ladanyi M (2009). Integrative clustering of multiple genomic data types using a joint latent variable model with application to breast and lung cancer subtype analysis. Bioinformatics.

[15] Ronglai S (2012). Integrative subtype discovery in glioblastoma using iCluster. Gynecol. Oncol..

[16] Zhang W (2013). Integrating genomic, epigenomic, and transcriptomic features reveals modular signatures underlying poor prognosis in ovarian cancer. Cell Rep..

[17] Wu D, Wang D, Zhang MQ, Gu J (2015). Fast dimension reduction and integrative clustering of multi-omics data using low-rank approximation: Application to cancer molecular classification. BMC Genom..

[18] Khan, A. & Maji, P. Selective update of relevant eigenspaces for integrative clustering of multimodal data. *IEEE Trans. Cybern.* 1–13 (2020). 10.1109/TCYB.2020.2990112 32452799

[19] Khan, A. & Maji, P. Approximate graph laplacians for multimodal data clustering. *IEEE Trans. Pattern Anal. Mach. Intell.* (2019). 10.1109/TPAMI.2019.2945574 31603770

[20] Xu T (2016). Identifying cancer subtypes from miRNA-TF-mRNA regulatory networks and expression data. PLoS One.

[21] Jiang L, Xiao Y, Ding Y, Tang J, Guo F (2019). Discovering cancer subtypes via an accurate fusion strategy on multiple profile data. Front. Genet..

[22] Long, B., Yu, P. S. & Zhang, Z. A General model for multiple view unsupervised learning. *In Proceedings of the 2008 SIAM International Conference on Data Mining* 822–833 (SIAM, 2008).

[23] Xia T, Tao D, Mei T, Zhang Y (2010). Multiview spectral embedding. IEEE Trans. Syst. Man. Cybern. Part B Cybern..

[24] Zhou, D. & Burges, C. J. Spectral clustering and transductive learning with multiple views. In *Proceedings of the 24th International Conference on Machine Learning* 1159–1166 (ACM, 2007).

[25] Zhang C (2020). Generalized latent multi-view subspace clustering. IEEE Trans. Pattern Anal. Mach. Intell..

[26] Li X, Zhang H, Wang R, Nie F (2022). Multiview clustering: A scalable and parameter-free bipartite graph fusion method. IEEE Trans. Pattern Anal. Mach. Intell..

[27] Gao Q (2021). Enhanced tensor RPCA and its application. IEEE Trans. Pattern Anal. Mach. Intell..

[28] Jha VN (2016). Study on Hermitian, Skew-Hermitian and unitary matrices as a part of normal matrices. Int. J. Open Inf. Technol..

[29] Collins, M., Dasgupta, S. & Schapire, R. E. A generalization of principal component analysis to the exponential family. In *NIPS’01: Proceedings of the 14th International Conference on Neural Information Processing Systems: Natural and Synthetic* 617–624 (2001).

[30] Schölkopf, B., Mika, S., Smola, A., Rätsch, G. & Müller, K.-R. Kernel PCA pattern reconstruction via approximate pre-images. In *International Conference on Artificial Neural Networks* 147–152 (Springer, 1998).

[31] Raykar, V. C. Spectral Clustering and Kernel Principal Component Analysis are Pursuing Good Projections. *Project Report* (2004).

[32] Schölkopf B, Smola A, Müller KR (1998). Nonlinear component analysis as a kernel eigenvalue problem. Neural Comput..

[33] Welling M (2005). Kernel principal components analysis. Adv. Neural. Inf. Process. Syst..

[34] Mantao, X. & Franti, P. A Heuristic k-means clustering algorithm by kernel PCA. In *2004 International Conference on Image Processing, 2004. ICIP ’04.*, vol. 5, 3503–3506 (2004).

[35] von Luxburg, U. A Tutorial on Spectral Clustering (2007). arXiv:0711.0189.

[36] Ng, A. Y., Jordan, M. I. & Weiss, Y. On spectral clustering: Analysis and an algorithm. In *Proceedings of the 14th International Conference on Neural Information Processing Systems: Natural and Synthetic*, NIPS’01, 849–856 (MIT Press, 2001).

[37] Gönen M, Alpaydın E (2011). Multiple kernel learning algorithms. J. Mach. Learn. Res..

[38] Network, T. R. Clinical significance of four molecular subtypes of gastric cancer identified by the Cancer Genome Atlas Project. *Clin. Cancer Res.* (2017). 10.1158/1078-0432.CCR-16-2211 PMC5785562 28747339

[39] Network TR (2017). Integrated genomic and molecular characterization of cervical cancers. Nature.

[40] Network TR (2015). Comprehensive, integrative genomic analysis of diffuse lower-grade gliomas. N. Engl. J. Med..

[41] Matsuno RK (2013). Agreement for tumor grade of ovarian carcinoma: Analysis of archival tissues from the surveillance, epidemiology and end results residual tissue repository. Cancer Causes Control.

[42] Huang, T., Yang, J. & Cai, Y. D. Novel candidate key drivers in the integrative network of genes, micrornas, methylations, and copy number variations in squamous cell lung carcinoma. *BioMed Res. Int.* (2015). 10.1155/2015/358125 PMC4352729 25802847

[43] Borel C (2011). Identification of cis- and trans-regulatory variation modulating microRNA expression levels in human fibroblasts. Genome Res..

[44] Lu J, Clark A (2012). Impact of microRNA regulation on variation in human gene expression. Genome Res..

[45] Liu F, Dong H, Mei Z, Huang T (2020). Investigation of miRNA and mRNA co-expression network in ependymoma. Front. Bioeng. Biotechnol..

[46] Dudziec E, Gogol-Döring A, Cookson V, Chen W, Catto J (2012). Integrated epigenome profiling of repressive histone modifications, DNA methylation and gene expression in normal and malignant urothelial cells. PLoS One.

[47] McMahon KW, Karunasena E, Ahuja N (2017). The roles of DNA methylation in the stages of cancer. PCancer J. (Sudbury, Mass.).

[48] Kim T, Jeong H, Sohn K (2019). Topological integration of RPPA proteomic data with multi-omics data for survival prediction in breast cancer via pathway activity inference. BMC Med. Genom..

[49] Zwiener I, Frisch B, Binder H (2014). Transforming RNA-seq data to improve the performance of progonostic gene signatures. PLoS One.

[50] Sun Y, Ou-Yang L, Dai D-Q (2021). WMLRR: A weighted multi-view low rank representation to identify cancer subtypes from multiple types of omics data. IEEE/ACM Trans. Comput. Biol. Bioinf..

[51] Wilkerson MD, Hayes DN (2010). ConsensusClusterPlus: A class discovery tool with confidence assessments and item tracking. PLoS One.

[52] Cai M, Li L (2017). Subtype identification from heterogeneous TCGA datasets on a genomic scale by multi-view clustering with enhanced consensus. BMC Med. Genom..

[53] Xu T (2017). CancerSubtypes: An R/bioconductor package for molecular cancer subtype identification, validation and visualization. Bioinformatics.

[54] Cabassi, A. & Kirk, P. D. W. Multiple Kernel Learning for Integrative Consensus Clustering of Omic Datasets. *arXiv preprint* (2019). 10.1093/bioinformatics/btaa593 PMC7750932 32592464

[55] Brunet JP, Tamayo P, Golub TR, Mesirov JP (2004). Metagenes and molecular pattern discovery using matrix factorization. PNAS.

[56] Khan, A. & Maji, P. Multi-manifold optimization for multi-view subspace clustering. *IEEE Trans. Neural Netw. Learn. Syst.* 1–13 (2021). 10.1109/TNNLS.2021.3054789 33606638

[57] Rousseeuw PJ (1987). Silhouettes: A graphical aid to the interpretation and validation of cluster analysis. Comput. Appl. Math..

[58] Bezdek, J. C. & Pal, N. R. Cluster Validation with Generalized Dunn’s Indices. In *Proceedings 1995 Second New Zealand International Two-Stream Conference on Artificial Neural Networks and Expert Systems. IEEE Xplore* 190–193 (1995).

[59] Davies DL, Bouldin DW (1979). A cluster separation measure. IEEE Trans. Pattern Anal. Mach. Intell..

[60] Xie X, Beni G (1991). A validity measure for fuzzy clustering. IEEE Trans. Pattern Anal. Mach. Intell..

[61] de Souto, M. C. P. *et al.* A comparison of external clustering evaluation indices in the context of imbalanced data sets. In *2012 Brazilian Symposium on Neural Networks* (2012).

[62] Hubert LJ, Arabie P (1985). Comparing partitions. J. Classif..

[63] Qiang W, Yong D, Xinwang L, Qi L, Shijie L (2016). Multi-view clustering with extreme learning machine. Neurocomputing.

[64] Smyth, G. K. Limma: Linear models for microarray data. In *Bioinformatics and Computational Biology Solutions Using R and Bioconducter*, vol. **214**, 397–420 (Springer, 2005).

[65] Yu G, Wang L, Han Y, He Q (2012). clusterProfiler: An R package for comparing biological themes among gene clusters. OMICS J. Integr. Biol..

[66] Vlachos, I. S. et al. Deciphering microRNA function with experimental support. DIANA-miRPath v3.0. *Nucleic Acids Res.* **43**, W460–W466 (2015). 10.1093/nar/gkv403 PMC4489228 25977294

[67] Yu G, Wang LG, Yan G, He QY (2015). DOSE: An R/Bioconductor package for disease ontology semantic and enrichment analysis. Bioinformatics.

[68] Kanehisa M, Goto S (2000). KEGG: Kyoto encyclopedia of genes and genomes. Nucleic Acids Res..

[69] Paul, S. & Madhumita. RFCM3: Computational method for identification of miRNA–mRNA regulatory modules in cervical cancer. *IEEE/ACM Trans. Comput. Biol. Bioinform.* **17**, 1729–1740 (2020). 10.1109/TCBB.2019.2910851 30990434

[70] Agarwal SM, Raghav D, Singh H, Raghava G (2011). CCDB: A curated database of genes involved in cervix cancer. Nucleic Acids Res..

